# The p53-S100A2 Positive Feedback Loop Negatively Regulates Epithelialization in Cutaneous Wound Healing

**DOI:** 10.1038/s41598-018-23697-5

**Published:** 2018-04-03

**Authors:** Shin-Chen Pan, Che-Yu Li, Chia-Yi Kuo, Yi-Zih Kuo, Wei-Yu Fang, Yu-Hsuan Huang, Tzu-Chin Hsieh, Hung-Ying Kao, Yuan Kuo, Ya-Rong Kang, Wan-Chi Tsai, Sen-Tien Tsai, Li-Wha Wu

**Affiliations:** 1 Department of Surgery, Section of Plastic and Reconstructive Surgery, National Cheng Kung University Hospital, College of Medicine, National Cheng Kung University, Tainan, Taiwan Republic of China; 2 Institute of Molecular Medicine, College of Medicine, National Cheng Kung University, Tainan, Taiwan Republic of China; 3 Department of Otolaryngology, National Cheng Kung University Hospital, College of Medicine, National Cheng Kung University, Tainan, Taiwan Republic of China; 4 Institute of Basic Medical Sciences, College of Medicine, National Cheng Kung University, Tainan, Taiwan Republic of China; 5 Department of Biochemistry, School of Medicine, Case Western Reserve University, Cleveland, OH USA; 6 Institute of Oral Medicine, College of Medicine, National Cheng Kung University, Tainan, Taiwan Republic of China; 7 Department of Laboratory Science and Technology, College of Health Science, Kaohsiung Medical University, Kaohsiung, Taiwan Republic of China; 8 Department of Radiation Oncology, National Cheng Kung University Hospital, College of Medicine, National Cheng Kung University, Taiwan, Republic of China

## Abstract

The S100A2 protein is an important regulator of keratinocyte differentiation, but its role in wound healing remains unknown. We establish epithelial-specific *S100A2* transgenic (TG) mice and study its role in wound repair using punch biopsy wounding assays. In line with the observed increase in proliferation and migration of *S100A2*-depleted human keratinocytes, mice expressing human *S100A2* exhibit delayed cutaneous wound repair. This was accompanied by the reduction of re-epithelialization as well as a slow, attenuated response of *Mcp1*, *Il6*, *Il1β*, *Cox2*, and *Tnf* mRNA expression in the early phase. We also observed delayed *Vegfa* mRNA induction, a delayed enhancement of the Tgfβ1-mediated alpha smooth muscle actin (α-Sma) axis and a differential expression of collagen type 1 and 3. The stress-activated p53 tumor suppressor protein plays an important role in cutaneous wound healing and is an *S100A2* inducer. Notably, S100A2 complexes with p53, potentiates p53-mediated transcription and increases p53 expression both transcriptionally and posttranscriptionally. Consistent with a role of p53 in repressing NF-κB-mediated transcriptional activation, S100A2 enhanced p53-mediated promoter suppression of *Cox2*, an early inducible NF-κB target gene upon wound injury. Our study thus supports a model in which the p53-S100A2 positive feedback loop regulates wound repair process.

## Introduction

Wound healing is an essential regenerative process required for the maintenance of the barrier function of the skin upon injury. Epithelialization, an essential process during skin wound healing, starts several hours after injury. Such process is orchestrated by multiple cell types and many factors, incluing cytokines and chemokines.

The transcription factor p53, activated by various cellular stresses such as DNA damage, hypoxia and oncogenic activation^1–3^, plays an important role in cutaneous wound healing process. The expression of p53 is, however, suppressed during active cellular proliferation in the injured swine tissue^4^. Transient inhibition of p53 in mice by pifithrin-α accelerates early epithelialization and neovascularization of cutaneous wounds by promoting leukocyte recruitment, increasing cell proliferation, and reducing apoptotic cell death^5^, suggesting a crucial role of p53 in wound healing.

The calcium-binding protein S100A2 (formerly called CaN19 or S100L) is primarily expressed in the basal layer of normal human epidermis and hair follicles^6^. S100A2 is induced by p53^7,8^, and in turn interacts with p53 and modulates transcription of *p21*, a p53 target^9,10^. *S100A2* mRNA is expressed in cycling cells that undergo squamous differentiation, suggesting a role of S100A2 in regenerative differentiation^11^. Furthermore, the epidermal growth factor (EGF) induces *S100A2* expression in human keratinocytes^12^ and topical application of EGF enhances cutaneous wound healing^13^.

We previously showed that S100A2 exerted tumor suppression in oral cancer via reducing the expression of inflammation-related cyclooxygenase 2 (COX2)^14^, a factor that is induced during rat skin wound repair^15^. Administration of the COX2 inhibitor delayed re-epithelialization in the early phase of wound healing and inhibited angiogenesis^15^. Given the role of S100A2 in the maintenance of keratinocyte functions and in regulating *COX2* expression, we hypothesized that S100A2 is a key component in wound healing. However, no S100A2 homolog has been identified in mice. In this study, we established an S100A2 transgenic mouse line to test our hypothesis and further examined the intricate relationship between p53 and S100A2.

## Results

### Establising transgenic mice expressing human S100A2 in epithelial cells

**Figure 1:**
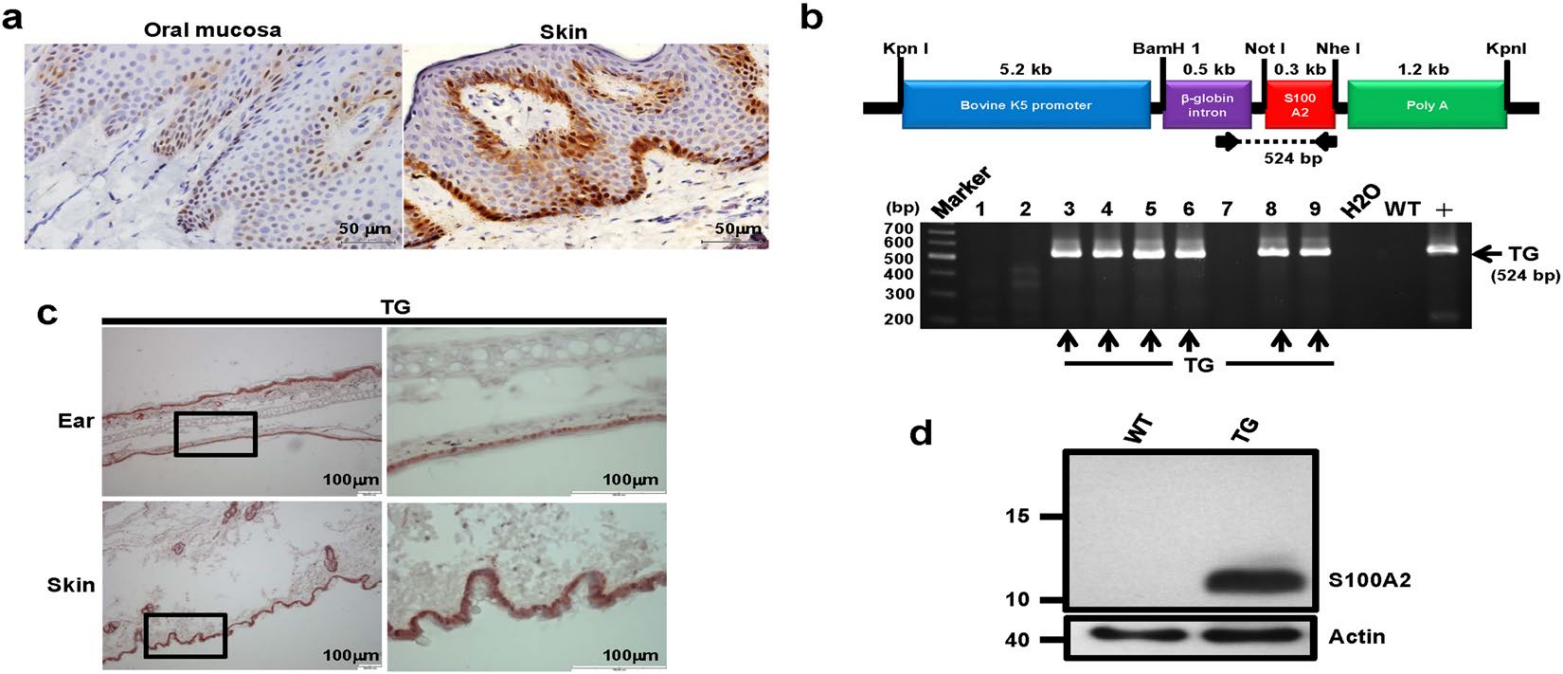
Expression of S100A2 in the skin of TG mice. (**a**) Representative immunohistochemical staining (400X magnification) of S100A2 in the basal cells of human oral mucosa (Left) or skin (Right). N = 2. (**b**) Top, A schematic representation of targeting vector for generating TG(K5-S100A2) mice. Bottom, Agarose gel analysis of PCR products derived from the mating progeny (lanes 1–9). Red arrows on the top panel are the primer pairs for genotyping S100A2 transgene allele in TG mice. (**c**) Expression of S100A2 protein in basal cells of TG mouse epidermis. Immunohistochemical staining of S100A2 in TG mouse ear (top) and skin (skin) are shown. Left panel, 100X magnification. Right panels, 400X magnification of insets in the left panels. (**d**) Western blot analysis of Flag-tagged S100A2 in TG mouse skin tissues using anti-S100A2 and actin (loading control) antibodies. Cropped Western blots are shown and full blots can be found in the supplementary information.

Immunohistochemical staining indicated that S100A2 was predominantly expressed in the basal layers in normal epithelium of oral cavity and human skin from two patients, regardless of tissue origin (Fig. 1a). Keratins are cytoplasmic intermediate filament proteins expressed in epithelial tissues in a site-specific and differentiation-dependent manner. Keratin 5 (K5) together with its partner keratin 14 (K14) are mainly expressed in mitotically active basal layers and their expressions are decreased as the cells undergo differentiation^16^. With the inability to detect S100A2 in mice^17,18^, we established TG(K5-S100A2) mice (Fig. 1b) in which the expression of Flag-tagged human S100A2 is driven by bovine K5 promoter in basal cells^17^, mimicking the predominant expression of S100A2 in basal cells of human epidermis. Immununohistochemical staining validated the basal cell expression of S100A2 in TG mouse ears and skin (Fig. 1c) but not in their kidney, liver or lung tissues (data not shown). In contrast, S100A2 expression was not detected in wildtype (WT) mouse ears and skin (Supplementary Fig. S1). Western blotting analysis of mouse skin protein lysates further confirmed the presence of human S100A2 in TG but not WT mice (Fig. 1d).

### Expression of epithelial S100A2 delays cutaneous wound healing in mice

**Figure 2:**
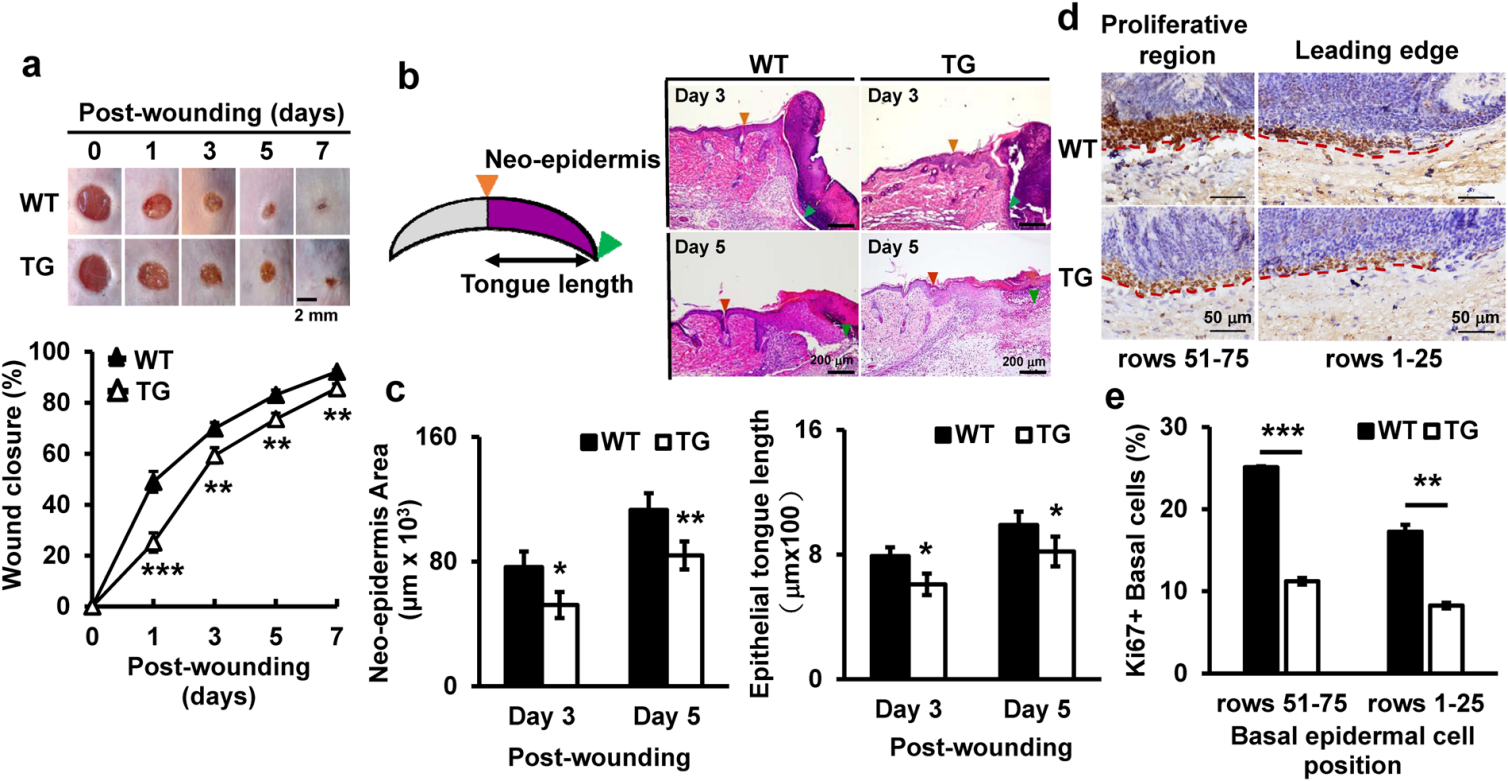
Delayed wound healing in S100A2 TG mice. Four full-thickness excision wounds were created on mouse dorsal skin by 4-mm biopsy punch. 5 mice per group. (**a**) Top, Representative images of wounds at different days post-wounding. Bottom, The graph shows the comparison of percent wound closure between TG and WT groups. (**b**) Left, A schematic for wound epithelium centered at the cut. Orange arrowhead, cut edge; green arrowhead, tongue tip. Right, representative H&E staining images in wounded skin on days 3 and 5 post-injury. (**c**) The quantification of neoepidermis (Left) and epidermal tongue length (Right) in HE staining sections on days 3 and 5 post-wounding. (**d**) The immunostaining of Ki67+ proliferating cells (brown nuclei) at day-3 wounds and (**e**) their mean (±s.e.m.) percentage in the indicated rows. Red dotted line, border for epidermis. Row numbers are counted from the tongue tip. *p < 0.05, **p < 0.01, ***p < 0.001.

Although S100A2 is mainly present in basal layer of the epidermis and hair follicles of normal skin^6^, its exact role in cutaneous wound repair is unclear. We determined the role of S100A2 in cutaneous wound healing by using 4-mm punch biopsy wounding model in TG and WT littermates. TG mice manifested a delay in the wound closure when compared to WT littermates (Fig. 2a). The quantification of hematoxylin and eosin (H&E) stained sections showed a reduction of neo-epidermis area and tongue length in TG mouse wounds at day 3 and day 5 (Fig. 2b,c). To examine if the reduction in wound healing was due to the decrease of re-epithelization, we scored the Ki67+ proliferating basal cells in the leading edges (rows 1–25) and proliferative regions (rows 51–75) at day 3 after establishing wound (Fig. 2d). TG mice exhibited significant decreases in the percentage of Ki67+ basal cells in wound areas (Fig. 2e). Based on these observations, we conclude that epithelial S100A2 expression delays wound healing, partly via reducing basal cell proliferation.

### S100A2 suppresses keratinocyte proliferation and migration

**Figure 3:**
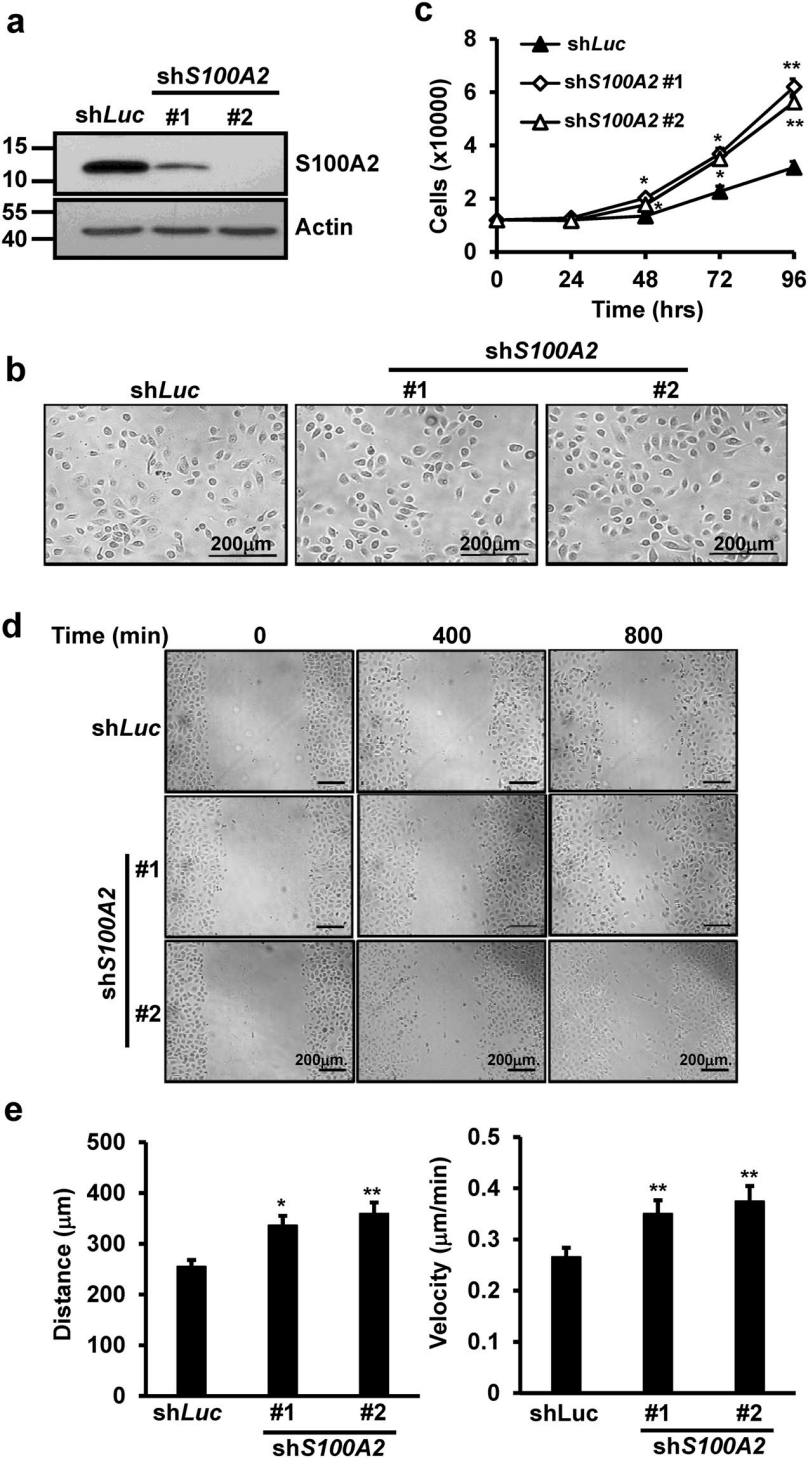
The knockdown of S100A2 expression promotes keratinocyte proliferation and migration. (**a**) Western blot analyses of S100A2 protein expression in control and *S100A2* knockdown aHK. Actin is used as a loading control. Cropped Western blots are shown and full blots can be found in the supplementary information. (**b**) The morphology of aHK bearing sh*Luc* control or sh*S100A2* taken under 200X magnification. (**c**) Proliferation rates of aHK bearing sh*Luc* control or sh*S100A2* clones (#1 and #2) were done in triplicates daily for 4 days. This result is a representative of 3 independent experiments. (**d**) The migration of sh*Luc* or sh*S100A2*-bearing aHK into the scratch wounds was measured by time-lapse video microscopy. Representative images were taken at 0, 400 and 800 min after the wounding. (**e**) Comparative measurements of distance migrated (Right) and velocity (Left) of the indicated cells in duplicates. This result is a representative of 2 independent experiments. Data represent mean ± s.d. ***p < 0.001, **p < 0.01, *p < 0.05 versus shLuc.

Re-epithelialization, often used as a defining parameter for a successful wound closure, involves proliferation and migration of keratinocytes^19^. Given the main presence of S100A2 in epidermis, we examined if S100A2 depletion alters keratinocyte proliferation and migration by using cell doubling and *in vitro* scratch wound assays. Two independent shRNAs (#1 and #2) were used to knock down S100A2 expression in adult keratinocytes (aHK) for clone establishment. Western blot analysis confirmed the successful depletion of endogenous S100A2 in both clones (Fig. 3a). No significant morphological change was observed in S100A2-depleted keratinocytes (Fig. 3b). However, a significantly increased aHK proliferation (Fig. 3c) and migration, manifested by migration distance and velocity (Fig. 3d,e), was observed in S100A2 knockdown cells, despite the lacking of a dose-dependent effect. Together, S100A2 negatively regulates keratinocyte proliferation and migration.

### Epithelial S100A2 reduces peak inflammation-related gene expression while delaying *Vegf-a* expression

**Figure 4:**
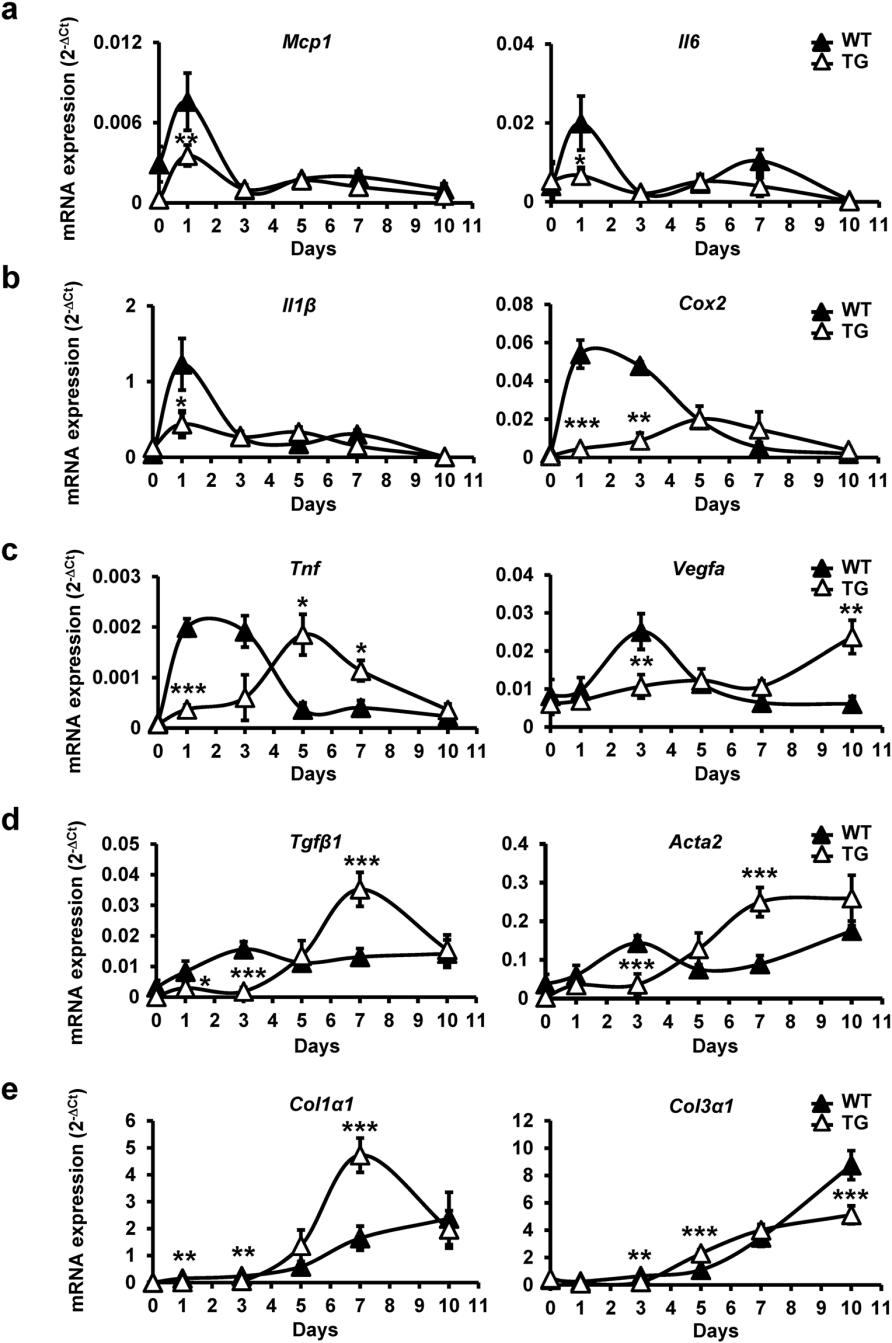
The expression profiles of signature genes involved in wound healing. qRT-PCR analysis of triplicates of the mRNA expression levels of the indicated genes post injury in WT and TG mice, 5 mice per group at each time point. (**a**) *Mcp1* and *Il6*. (**b**) *Il1β* and *Cox2*. (**c**) *Tnf* and *Vegfa*. (**d**) *Tgfβ1* and *Acta2*. (**e**) *Col1α1* and *Col3α1*. Triplicated data are represented as mean ± s.e.m. (N = 5). ***p < 0.001, **p < 0.01, *p < 0.05 versus +/+ at indicated time points.

Dermal wounding is accompanied by inflammation and the subsequent induction of proinflammatory cytokines released by keratinocytes, platlets or macrophages is thought to mediate the repair process^20–22^. The gene encoding cyclooxygenase 2 (COX2), a critical enzyme involved in the inflammatory response^23^, is induced in response to wound injury^24^. Angiogenic processes at the wound, especially the production of *VEGFA*, also contribute to normal skin repair^25^. To elucidate the mechanism underlying S100A2-mediated wound delay, we determined the expression profiles of *Vegfa*, and proinflammatory genes, including *Mcp1*, *Il6*, *Il1β*, *Tnf*, and *Cox2* mRNA in TG mice. The mRNA expression of *Mcp1*, *Il6*, *Il1β*, *Tnf* and *Cox2* peaked at day 1–2 while that of *Vegfa* peaked at day 3 post-wounding in WT mice (Fig. 4a–c). Notably, the peak of *Mcp1*, *Il6*, and *Il1β* expression at day 1 was significantly reduced in TG mice (Fig. 4a,b). Furthermore, the reduced peak *Cox2* expression was delayed until day 5 post wounding in TG mice (Fig. 4b, Right). We also observeded a delayed peak induction of *Tnf* and *Vegfa*, respectively, at day 5 and day 10 post injury in TG mice (Fig. 4c). These data indicated that epithelial expression of human S100A2 reduced or delayed the expression of mouse inflammatory genes and *Vegfa*.

### Epithelial S100A2 enhances TGFβ1-α-SMA signaling axis but differentially regulates the expression of collagen subtypes

Fibroblast-to-myofibroblast differentiation is a key event for tissue repair. TGFβ1 is an essential trigger promoting myofibroblast formation^26^. Alpha smooth muscle actin (α-SMA) encoded by *ACTA2* is a marker for myofibroblasts and mature vascular blood vessel cells^26^. We found that there was a concordant induction of both *Tgfβ1* and *Acta2* mRNAs at day 3 post-wounding in WT mice. Interestingly, their peak expressions were significantly elevated by >2 folds in TG mice (Fig. 4d), though delayed until day 7 post-wounding.

Collagen is fundamental for the contiguous formation of the interstitium through the epidermis. Collagen type I (Col1) and III (Col3) are the most abundant subtypes in skin^27^. Consistent with the notion, the expression of *Col1α1* and *Col3α1* increased with recovery time and peaked at day 10 post-injury in WT mice. Epithelial S100A2 expression induced 3-fold increase in peak *Co1α1* expression at day 7 but a 2-fold decrease in *Co13α1* expression at day 10 post-wounding (Fig. 4e). Collectivelly, epithelial S100A2 delayed but enhanced the peak induction of TGFβ1-α-SMA signaling axis while differentially regulating collagen subtype expression during cutaneous wound healing.

### S100A2 potentiated the transcriptional activity of p53

**Figure 5:**
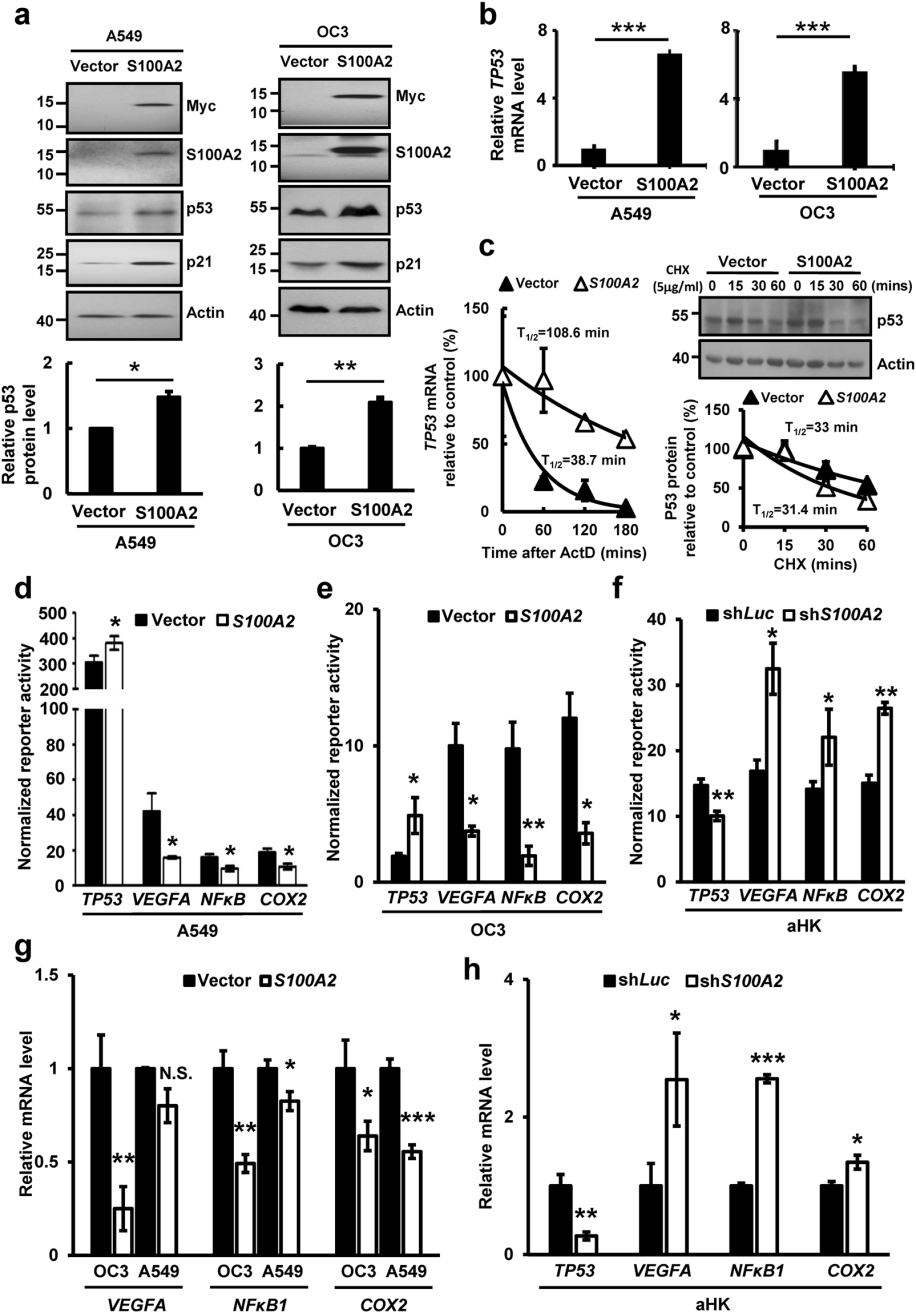
S100A2 potentiated the transcriptional activity of p53. (**a**) Western blot analysis of A549 (Left) and OC3 (Right) cells stably expressing vector or *S100A2*. Cropped Western blots are shown and full blots can be found in the supplementary information. (**b**) The effects of S100A2 on *TP53* mRNA expression. Total RNA was extracted from S100A2 expressing or control cells followed by RT-qPCR analyses. (**c**) The effects of S100A2 on *TP53* mRNA (Left) and protein (Right) stability following actinomycin D (ActD) or cyclohexmide (CHX) treatment. Cropped Western blots for p53 protein stability are shown and full blots can be found in the supplementary information. (**d**,**e**) The effects of overexpressing S100A2 on the activity reporter driven by promoters of *TP53*, *VEGFA*, synthetic *NFKB1* and *COX2* in A549 or OC3 cells. (**f**) The effects of *S100A2* knockdown (clone #2) on the reporter activity driven by the promoters of *TP53*, *VEGFA*, *NFKB1* and *COX2* in aHK. (**g**) The effects of overexpressing S100A2 on *COX2*, *NFKB1* and *VEGFA* mRNA expression in A549 or OC3 cells. (**h**) The effect of *S100A2* knockdown on *TP53*, *VEGFA*, *NFKB1*,and *COX2* mRNA expression in aHK. This result is a representative of three independent experiments, each performed in triplicates. Data are represented as mean ± s.d. *p < 0.05, **p < 0.01, ***p < 0.001.

S100A2 interacts with p53 and potentiates the transcriptional activity of p21, a p53 target^9^. We confirmed the interaction of S100A2 with p53 in aHK by reciprocal immunoprecipitation and promixity ligation assay (Supplementary Fig. S2). To avoid the confounding factor from *TP53* mutations, we instead used 2 cell lines derived from oral and lung cancer, OC3^28^ and A549^29^, both of which express wildtype *TP53*. Although at a lower level, both cell lines also expressed *S100A2* mRNA when compared to aHK (Supplementary Fig. S3). Remarkably, ectopically expressed S100A2 increased p53 and p21 protein as well as mRNA expression in OC3 or A549 cells, further supporting the presence of functional p53 in these cells. The extent of p53 protein induction was, however, not as distinct as that at mRNA level (Fig. 5a,b), indicating a possible role of S100A2 in regulating both mRNA and protein levels. To dissect the mechanisms underlying S100A2-induced p53 protein accumulation, we treated the indicated cells with actinomycin D for transcription blockage or with cycloheximide for de novo protein synthesis inhibition. Ectopically expressed S100A2 increased p53 expression in OC3 cells via increasing p53 mRNA rather than protein stability (Fig. 5c).

p53 suppresses the expression of NFκB, a master inflammation regulator and a transcription activator of *VEGFA* and *COX2*^30–33^. To confirm if S100A2 potentiated p53-mediated transcriptional repression of these 3 genes, we carried out transient transfection reporter assays in OC3 and A549 cells stably expressing vector or Myc-tagged S100A2 using the indicated gene promoter-driven luciferase constructs. Indeed, stably expressed S100A2 activated the reporter activity of the native *TP53* promoter, while reducing the reporter activities of native *VEGFA* and *COX2* promoters as well as the synthetic *NFκB* promoter (p2X-*NFκB*-Luc) (Fig. 5d,e). Additionally, *S100A2* knockdown had the opposite effect on these promoter activities in aHK, which express endogenous *S100A2* (Fig. 5f). *NFKB1* encodes p65, a subunit of the cannonical NFκB transcription factor known to play a role in inflammatory response^34^. Lastly, the expression of endogenous *NFKB1*, *VEGFA*, and *COX2* mRNAs was also reduced in OC3 and A549 cells overexpressing *S100A2*, with the exception of *VEGFA* expression in A549 cells (Fig. 5g). Moreover, *S100A2* depletion decreased *TP53* mRNA expression, while increasing the mRNA expression of *NFKB1*, *VEGFA* and *COX2* in aHK (Fig. 5h). We conclude that S100A2 represses the expression of *NFKB1, VEGFA*, and *COX2* mRNAs.

### S100A2 potentiates p53-mediated suppression of NFκB-induced *COX2* promoter activity

**Figure 6:**
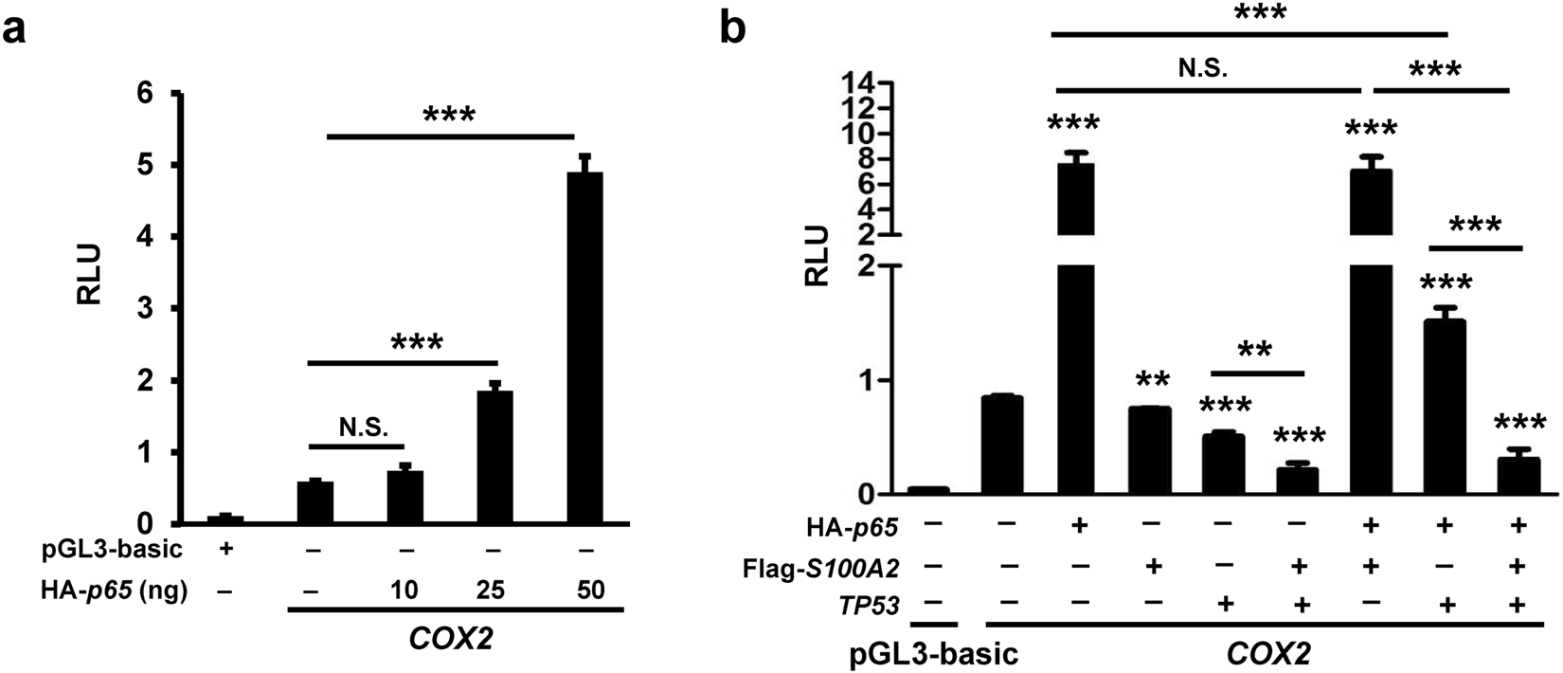
The effects of S100A2 on p53-mediated inhibition of NFκB1-induced *COX2* promoter activity. (**a**) The effects of HA-p65 on native *COX2* promoter reporter activity in p53-null H1299 cells. (**b**) The effects of of S100A2 on p53-mediated inhibition of NFκB1-induced *COX2* promoter activity. This result is a representative of three independent experiments, each performed in triplicates. Normalized reporter activity was presented as mean ± s.d. **p < 0.01, ***p < 0.001, N.S., not significant.

NFκB transcriptionally activates *COX2* expression^35^, whereas p53 inhibits this induction^31,36^. The expression of *COX2* is induced following epidermal injury^37^. We found that S100A2 suppresses *COX2* promoter activity in functional p53-expressing cells (Fig. 5g). Because S100A2 has no known DNA-binding activity and interacts with p53, we hypothesize that S100A2 suppresses *COX2* expression in a p53-dependent manner. We first demonstrated that HA-p65 activates *COX2* promoter activity in a dose-dependent manner in *TP53*-null H1299 cells (Fig. 6a). Importantly, the ability of S100A2 to inhibit HA-p65-induced *COX2* promoter activity depended on the presence of p53 (Fig. 6b). Collectively, S100A2 potentiated the p53-mediated transcriptional repression of *COX2* expression.

## Discussion

In this study, we demonstrated that epithelial S100A2 expression delayed wound healing in punch biopsy-induced wound model. The delay in TG mice was accompanied by reduced and/or delayed expression of *Vegfa*, inflammation-related genes and the Tgfβ1-α-Sma signaling axis as well as distinct regulation of collagen subtype expression during the healing process. Gene silencing studies in human skin cells further showed that *S100A2* depletion significantly promoted keratinocyte proliferation and migration. S100A2 formed a complex with p53 *in vitro* and *in vivo*. Forced S100A2 expression increased p53 expression as well as p53-mediated transcription. Together, epithelial S100A2 negatively regulated wound repair through potentiating p53-mediation in keratinocytes.

Keratinocytes are responsible for restoring the epidermis after injury through re-epithelilization^38^. In this process, keratinocytes migrate to denuded area and proliferate in response to multiple regulators. The cutaneous wound repair delay in TG mice was manifested by the decrease of neoepidermis area and tongue length as well as the reduction of Ki67-positive basal cells when compared with WT littermates (Fig. 2). Our observation of the enhancement of human keratinocyte migration and proliferation induced by *S100A2* depletion further supports a negative role of S100A2 in re-epithelization.

Cytokines are critical mediators for cell recruitment in the early stage of wound healing. The induced expression of proinflammatory Il6, Il1β and Tnf cytokines were detected as early as 12–24 hours after cutaneous injury^39,40^. They affect various processes of healing including the stimulation of keratinocytes proliferation^41^. Although early induction of these genes is important for normal repair, the induction of *Il6*, *Il1β* and *Tnf* was significantly reduced in TG mice. Macrophages are one of the immune cells critical for wound repair^22^ and can be recruited by MCP1 produced by monoctyes, keratinocytes and many other cell types^42,43^. The significant reduction of *Mcp1* at day 1 post injury (Fig. 4a) was followed by the decrease of macrophage marker *F4/80* expression at day 7 (data not shown), in TG mice. Overall, the inflammatory activity of S100A2-expressing wounds was markedly reduced in early stage of wound repair compared with WT controls.

The increase in the density of blood vessels compared to uninjured skin is commonly observed during the proliferative phase of healing as a result of angiogenesis. VEGFA is one of the most potent pro-angiogenic factors in the skin, and its amount present in a wound can impact the healing. Both keratinocyte-derived and myeloid cell–derived VEGFA affect the repair process^44^. Reducing VEGFA activity by treating inhibitors for VEGF signaling or conditional genetic silencing of *VEGFA* delayed wound healing^45,46^. Consistent with the reduced levels of VEGFA in the delayed healing wounds, we also detected a delayed induction of *Vegfa* in the TG mouse wounds (Fig. 4c, right panel).

The high contractile force generated by myofibroblasts is beneficial for physiological tissue remodeling but detrimental to tissue function when becoming excessive such as that in hypertrophic scars^26^. TGFβ1, required for the fibroblast-to myofibroblast differentiation, drastically induces α-SMA expression in granulation tissue myofibroblasts and cultured fibroblasts^47,48^. Consistent with this observation, there was a concordant induction of both *Tgfb1* and *Acta2* at day 3 in WT mice after cutaneous injury (Fig. 4d). Although TGFβ1 is involved in wound healing with contrasting roles^41^, the epidermal expression of *Tgfb1* driven by *K14* promoter delayed re-epithelialization via inhibiting keratinocyte proliferation in both burn and full-thickness excisional wound models^49,50^. The wound repair delay detected in TG mice was aslo accompanied with the delayed peak induction of Tgfβ1-α-Sma axis at day 7–10 post wounding (Fig. 4d), reminiscence of the delayed epithelialization detected in the *K14*-driven *Tgfb1* TG mice. More studies are warranted to address the action mechanism whereby epithelial S100A2 delayed the induction of Tgfβ1-α-Sma signaling axis and consequently fibroblast activation during wound repair.

The remodeling phase of cutaneous wound usually initiates approximately at 9–10 days following injury in mice^51^. Collagen is the main extracellular matrix component during the remodeling and mediates the strength and elasticity of healthy skin and scar tissue. Two major subtypes, type I and type III, can be identified in normal skin with type I being the predominant type. Excessive type I collagen secretion results in disorganized fiber structure and hypertrophic scar formation^27^. Type III collagen was expressed both in the early stage of granulation tissue formation^52^ and late stage of scar hypertrophy^53^. Moreover, a significant increase of type I and III pro-collagen mRNA was found in burn scar tissue formation^54^. Since a differential expression of collagen I and III was noted between TG and WT mice, the influence of epithelial S100A2 on collagen synthesis and scar formation should be further validated.

S100A2 interacts with p53 *in vitro* and *in vivo*. S100A2 was previously shown to induce *p21* promoter activity in reporter assays^9^. We showed that S100A2 potentiates the ability of p53 to repress the mRNA expressions of *NFKB1, VEGFA* and *COX2* (Fig. 5g,h). Unexpectedly, *TP53* mRNA and protein levels are elecvated in S100A2-overexpressing cells (Fig. 5a,b), allthough no difference was observed on p53 protein turnover in control or S100A2 overexpressed cells (Fig. 5c). Indeed, S100A2 transcriptionally activates *TP53* promoter activity (Fig. 5d,e) and stabilizes *TP53* mRNA (Fig. 5c). Although S100A2 was also shown to interact *in vitro* with p63, a p53 homolog^10^, we did not observe concordant changes of p63 protein expression in response to overexpression of *S100A2* (Supplementary Fig. S4). We concluded that the ability of S100A2 to potentiate p53 activity occurs at two levels: (1) S100A2 increases p53 protein expression by inducing its mRNA accumulation and (2) S100A2 potentiates p53 transcriptional activity through its physical interaction.

The inflammatory activity of S100A2-expressing wounds was markedly reduced relative to their WT littermates. NFκB regulates the expression of *TNFα*, *IL1β*, *IL6* and *MCP1*^39,40,55^. The activation also plays a key role in VEGFA production by macrophages^56^ and controls *COX2* transcription^57^. The induced *COX2* expression coincided with keratinocyte proliferation in epidermal repair^15,58^. The inhibition of COX2 activity delayed re-epithelialization and angiogenesis during cutaneous wound healing. S100A2 suppressed *COX2* expression in oral cancer cells^14^. We also detected a delay in the reduced peak expression of *Cox2* in TG mice (Fig. 4b). The compromised expression of several inflammatory cytokines as well as *Cox2* in TG mice suggests the involvement of NFκB activity in S100A2-mediated wound delay. S100A2, however, had no direct effect on modulating the activity of NFκB (Fig. 6b).

Although S100A2 is a distinct S100 family member with nuclear localization^59^, several studies reported the detection of S100A2 in lung cancer patient sera and the negative effect of *S100A2* overexpression on squamous cell carcinoma migration^60,61^. We also detected the presence of S100A2 protein in the cultured keratinocyte medium (data not shown). At least two receptor populations including the receptor for advanced glycation end-products (RAGE) were suggested to act as the receptors for extracellular S100A2^60,62^. In addition to the intracellular effects of S100A2, the extracellular effect on the cells expressing the receptors for S100A2 might also account for its effect on the delayed and/or reduced expression of several inflammation-related genes as well as angiogenic *VEGFA* in the healing wounds.

In conclusion, epithelial S100A2 inhibited skin re-epithelialization after punch wound injury. In addition to the direct modulatory effect of S100A2 on basal epithelial cell proliferation and migration, the delayed re-epithelialization in TG mice could also be accounted for by a slow acting response of cytokine expression and angiogenesis in the early stage of wounding. Forced *S100A2* expression enhanced *TP53* expression via both transcriptional and post-transcriptional regulation. Since both *TP53* and *NFKB1* expression was differentially induced during mouse wound repair (Supplementary Fig. S5)^4,63^, the complexity of p53 crosstalk with NFκB added another layer of complexity in the S100A2-mediated regulation of wound repair. Epithelial S100A2 may be a potential target for manipulating the wound healing process.

## Materials and Methods

### Materials

The source and the use of all the antibodies were listed in Supplementary Table S1. Culture media, fetal bovine serum (FBS), Lipofectamine 2000, TRIzol and qRT-PCR reagents were from ThermoFisher Scientific (Waltham, MA, USA). Oligonucleotide primers for sequencing and qRT-PCR were from MDbio (Taipei, Taiwan). Dual Luciferase reporter assay were from Promega (Madison, WI, USA).

### Clinical specimens

After obtaining appropriate institutional review board permission and informed consent from the patients receiving the treatment, the oral mucosa specimens were from the individuals with crown lengthening procedure for dental restoration. The normal skin sample was obtained from the right thigh of patients who underwent reconstructive surgery. Their use for Immunohistochemistry analysis was previously approved by the Institutional Review Board at National Cheng Kung University Hospital. All experiments of using clinical specimens were performed in accordance with relevant guidelines and regulations.

### Mice

The targeting vector, pBK-Flag-S100A2, for making S100A2 transgenic mice^17^ was generously provided by Dr JT Elder at University of Michigan at Ann Arbor. The transgenic mice, TG(K5-S100A2), and their littermates were bred in National Laboratory Animal Center in Taiwan. We performed genotyping by using tail snip DNA and primers listed in Supplementary Table S2. Three founders were obtained and bred to F4-5 generation. All the use of these animlas and experimental protocols were reviewed and approved by the Institutional Animal Care and Use Committee (IACUC), National Cheng Kung University. All methods were performed in accordance with the relevant guidelines and regulations.

### Punch biopsy

The 6- to 7-week-old male mice were anesthetized and the dorsal hairs were removed by depilatory creams. Four full-thickness excision wounds were punched at two sites in the middle of the dorsum using a diameter of 4-mm biopsy punch. The punched out skin served as day 0. Each wound region was digitally photographed at the indicated time points. The wound area was quantified using Image J software 1.48 (National Institute of Health, Bethesda, MD, USA). The wounds were left uncovered and harvested using a 6-mm biopsy punch at the indicated time points, five mice per time point. Percent wound closure {[1 − (wound area)/(original wound area)] × 100%} was calculated at days 0, 1, 3, 5 and 7.

### Immunohistochemistry and histological analysis

The wounded skins at indicated time points were fixed with 10% formalin, embedded in paraffin, and cut into 4-μm-thick sections. The sections were deparaffinized three times in xylene, 5 minutes each, and rehydrated through graded ethanol solutions. Antigen retrieval was carried out by steam heating for 20 minutes in 0.01 M citrate buffer (pH 6.0). The fixed sections were stained with H&E stain or the antibodies of interest as shown in Table S2. We detected the immunocomplex by Dako REAL™ EnVision™ Detection System, Peroxidase/DAB+ (Hamburg, Germany) and quantified the stained area by using Image J software 1.48.

### Culture and establishment of S100A2-manipulated cells

Human epidermal keratinocytes from adults, aHK, were purchased from and propagated as described by ThermoFisher Scientific (Waltham, MA, USA). H1299 and 293 T cell lines were cultured in DMEM with 10% heated-inactivated FBS, and penicillin/streptomycin. Lung adenocarcinoma A549 cells were propagated in MEM with 10% heat-inactivated FBS, 1 mM sodium pyruvate, non-essential amino acids, and penicillin/streptomycin. Squamous cell carcinoma OC3 cells were maintained as described^28^. For shRNA-lentiviral preparation, control sh*Luc* or sh*RNA* clones to human *S100A2* (clone #1: TRCN0000053539; clone #2: TRCN0000053538) were transfected into HEK-293T cells by using Lipofectamine 2000. Following collection of viral particles at 48 hours post transfection, their titers were measured by using endpoint dilution assay that quantifies the amount of viruses required to kill 50% of infected A549 cells. The indicated cells were infected with the viruses at multiplicity of infection (MOI) at two for 24 hours. Stable clones were enriched for 72 hours with 1 μg/ml puromycin post-infection. As to ectopic S100A2-expressing stable clones, pcDNA3.1 control or pcDNA3.1-Myc-S100A2 plasmids were transfected into the indicated cells by using Lipofectamine 2000. S100A2-stable expressing cells were established with 1 mg/ml G418 selection for at least 2 weeks.

### Western blot analysis

Total protein was extracted from the indicated cells by using boiled SDS lysis buffer or from mouse tissues by using ice-cold lysis buffer of 50 mM Tris-HCl (pH 8), 150 mM NaCl, 2 mM EDTA (pH 8), and 0.2% NP-40 supplemented with a protease inhibitor cocktail (Roche, Basel, CH). Equal amounts of total protein were fractioned by SDS-PAGE and blotted onto polyvinylidene difluoride membrane. The protein blots were probed with indicated primary and then secondary antibodies. The hybridized immune complex was detected by Immobilon Western Chemiluminescent HRP substrate (Darmstadt, Germany).

### Cell doubling

Cells were seeded at a confluency of 20–30% in 24-well plates. Following the use of trypan blue exclusion assay, viable cells were enumerated daily for 4 days after seeding.

### *In vitro* scratch wound assay

The cells were transfected with the indicated plasmid DNA or infected with the indicated shRNA-bearing lentiviruses before the wounding. The indicated cells were seeded at a density of 90% in 6-well plates coated with 5 μg/ml collagen. Following seeding for 24 hrs, confluent cells were fed with serum-reduced growth media (2% FBS) containing mitomycin C (1 μg/ml) for 24 hrs. We scratched the monolayer with 200-μl pipette tips to generate 3 parallel scratches. Following saline washes, the wound was photographed by time-lapse video microscopy for 800 min in 5% CO_2_, 37 °C incubator. Individual keratinocyte migration track (≧30 cells) was measured by ImageJ software. The migration distance (μm) and velocity (μm/min) of ≧30 cells were scored and calculated as described^64^.

### qRT-PCR

Total RNA was extracted from snap-frozen skin samples using TRIZOL reagent. One μg sample of total RNA was reverse-transcribed into cDNA in a volume of 20 μl with random hexamers, oligo dT primer and MMLV reverse transcriptase (Promega, Madison, WI, USA). Real-time PCR in triplicate was performed by using StepOne™ Real-Time PCR System (Applied Biosystems, Foster City, CA, USA). The relative expression of mRNA was calculated using the 2^−ΔCt^ method with GAPDH or 28S rRNA as a reference gene. The primers for real-time PCR were listed in Supplementary Table S2.

### Drug treatment

For the transcriptional inhibition, cells were exposed for 1 hr with actinomycin D (5 μg/ml) and allowed to recover for the indicated time prior to RNA isolation for qRT-PCR analysis. As to the inhibition of de novo protein synthesis, cells were treated for the indicated time with cycloheximide (2 μg/ml) prior to total protein harvest for Western blot analysis.

### Luciferase assay

The *Cox2* proximal promoter (−1432/+59) was provided as described^65^. We cloned human *VEGFA* (−1231/+1094) and TP53 (−2063/+160 based on NM_000546.5) promoters^66^ using transcription start as +1 into pGL3 basic vector. Synthetic 2X-*NFκB*-Luc, a generous gift of Dr. Karin M, was used for measuring *NFκB* promoter activity. After seeding in 24-well plates for 16–18 hrs, the indicated cells were seeded in triplicate and transiently transfected with indicated plasmids for 6 hours by using Lipofectamine 2000. Forty-eight hours after transfection, the luciferase activity in lysates was measured by using Dual-Luciferase^®^ reporter assay (Promega, Madison, WI, USA) and expressed as relative luciferase units (RLU). Renilla luciferase activity was an internal control for transfection efficiency. The indicated promoter activity was expressed as normalized reporter activity following the normalization of the indicated promoter RLU with promoterless pGL3-basic vector RLU.

### Statistical analysis

Statistical analyses were performed using one way analysis of variance (ANOVA) to compare all pairs of experimental groups. Data were represented as mean ± s.d. or s.e.m. p < 0.05 was regarded as statistically significant. For comparison of two groups, two-tailed student’s t tests were used. Statistical significance was defined as P < 0.05.

### Data availability

All data generated or analysed during this study are included in this published article (and its Supplementary Information files). Raw datasets generated are available from the corresponding author on reasonable request.

## Electronic supplementary material

Supplementary information
