# Supplementary material for: The p53-S100A2 Positive Feedback Loop Negatively Regulates Epithelialization in Cutaneous Wound Healing
Source: Sci Rep. 2018 Apr 3;8:5458. doi: 10.1038/s41598-018-23697-5 (PMC5882638; doi:10.1038/s41598-018-23697-5)

The p53-S100A2 Positive Feedback Loop Negatively Regulates Epithelialization in Cutaneous Wound Healing

Shin-Chen Pan[1], Che-Yu Li[2], Chia-Yi Kuo[2], Yi-Zih Kuo[3], Wei-Yu Fang[4], Yu-Hsuan Huang[2], Tzu-Chin Hsieh[2], Hung-Ying Kao[5], Yuan Kuo[6], Ya-Rong Kang[2], Wan-Chi Tsai[7], Sen-Tien Tsai[3,8,*], and Li-Wha Wu[2,7,*]

[1]Department of Surgery, Section of Plastic and Reconstructive Surgery, National Cheng Kung University Hospital, College of Medicine, National Cheng Kung University, Tainan, Taiwan, R.O.C.

[2]Institute of Molecular Medicine, College of Medicine, National Cheng Kung University, Tainan, Tainan, Taiwan, R.O.C.

[3]Department of Otolaryngology, National Cheng Kung University Hospital, College of Medicine, National Cheng Kung University, Tainan, Tainan, Taiwan, R.O.C.

[4]Institute of Basic Medical Sciences, College of Medicine, National Cheng Kung University, Tainan, Tainan, Taiwan, R.O.C.

[5]Department of Biochemistry, School of Medicine, Case Western Reserve University, Cleveland, OH, U.S.A.

[6]Institute of Oral Medicine, College of Medicine, National Cheng Kung University, Tainan, Tainan, Taiwan, R.O.C.

[7]Department of Laboratory Science and Technology, College of Health Science, Kaohsiung Medical University, Kaohsiung, Taiwan, R.O.C.

[8]Department of Radiation Oncology, National Cheng Kung University Hospital, College of Medicine, National Cheng Kung University, Tainan, Taiwan, R.O.C.

*, Corresponding authors

Correspondence:

Li-Wha Wu, Institute of Molecular Medicine, College of Medicine, National Cheng Kung University, 1 University Road, Tainan 70101, Taiwan, R.O.C.; Tel.: +886-6-2353535 ext.3618; Fax: +886-6-2095845; E-mail: liwhawu@mail.ncku.edu.tw.

Sen-Tien Tsai, Department of Otolaryngology, National Cheng Kung University Hospital, 138 Sheng-Li Rd., Tainan 70428, Taiwan, R.O.C. Tel:+886-6-2353535 ext.5311; E-mail: T602511@mail.ncku.edu.tw.

Supplementary information

Methods

Immunoprecipitation

The indicated transfected or infected cells were extracted by ice-cold lysis buffer of 50 mM Tris-HCl (pH 8), 150 mM NaCl, 1% NP-40, 0.1% SDS and 0.5% sodium deoxycholate supplemented with a protease inhibitor cocktail. For p53 immunoprecipitation, the indicated cells were incubated with an anti-p53 monoclonal antibody (1:500 dilution) overnight at 4℃ by using Catch and Release® v2.0 Reversible Immunoprecipitation System (Millipore Corp., Billerica, MA, USA). For immunoprecipitation of Flag-tagged S100A2, 500 μg of protein lysates were incubated with 10 μl Anti-DYKDDDDK (Flag) Affinity Gel overnight at 4℃. Following three washes with the lysis buffer, the immunocomplexes were fractionated

and detected by using Western blot analysis.

Proximity ligation assay

Duolink® In Situ was performed according to the manufacturer's instructions (Sigma-Aldrich, St. Louis, MO, USA). Briefly, aHK and OC3 cells grown on coverslips were fixed in 4% paraformaldehyde for 10 min before 10-min permeabilization in PBS containing 0.5% Triton X-100. Following blocking in 1% bovine serum albumin, the fixed cells were incubated with primary antibodies to S100A2 and to p53. After cell washing, PLA probes were added, followed by hybridization, ligation, and amplification for 100 min at 37℃. DNA (blue) and S100A2-p53 interaction (red) were visualized after incubation with the Detection solution. Slides were analyzed by fluorescence microscopy.

Supplementary Tables and Figures

Supplementary Table S1. Antibodies and their use for experiments

Supplementary Table S2. Primer list

Legend to Supplementary Figures

Supplementary Figure S1. S100A2 was not found in WT mice.

Immunohistochemical staining of S100A2 in WT mouse ear (top) and skin (skin) are shown. Left panels, 100X magnification. Right panels, 400X magnification of insets in the left panels.

Supplementary Figure S2. p53 and S100A2 directly bind to each other

(a) The interaction of endogenous p53 and S100A2 was validated in aHK cells by reciprocal immunoprecipitation and Western blot analysis. Isotypic IgG is a negative control. Cropped Western blots are shown and full blots can be found in the supplementary information. (b) The interaction of endogenous p53 and S100A2 was

also confirmed by proximity ligation assay (PLA). Blue, DAPI stain for nuclei. PLA, red stain. Merge, DAPI and PLA. This experiment was independently repeated two times.

Supplementary Figure S3. Differential S100A2 mRNA expression in aHK, OC3 and A549. qRT-PCR analysis of triplicates of the mRNA expression levels of S100A2 in the indicated cells. Triplicated data are represented as mean ± s.d. (N=2).

Supplementary Figure S4. The expression of p63 in S100A2-manipulated cells. Protein lysates were isolated from the S100A2-overexpressing OC3 (Left) or A549 (Middle) cells as well as S100A2-depleted aHK (Right). The expression of S100A2, p63 and actin (loading control) was detected by the indicate antibodies. Cropped Western blots are shown and full blots can be found in the supplementary information.

Supplementary Figure S5. The differential expression of *Tp53* and *Nfκb1* mRNA during mouse cutaneous wound repair

Total RNA was extracted from skin wound at indicated time points and reverse transcribed into cDNA. We performed qRT-PCR analysis in triplicate of the expression level of *Tp53* (Top) and *Nfκb1* (Bottom) mRNA. The relative mRNA expression was calculated using 2 delta CT method with Gapdh as the loading control. Data represent mean ± s.e.m. (N=5). *p<0.05; **p<0.01; p<0.001 versus day 0 wounds.

Supplementary Table S1 Antibodies and their use for experiments

Antibody	Source	Clone	Dilution	Experiment name
Anti-S100A2	BD Biosciences	14	1:1000	Western blot
Anti-S100A2	Abcam		1:500 /<br>1:200 /<br>1:50<br>1:100	Western blot /<br>Immunohistochemistry /<br>Immunoprecipitation<br>Proximity ligation assay
Anti-Myc	Thermo Fisher Scientific	Myc.A7	1:5000	Western blot
Anti-p53	Cell signaling Technology	1C12	1:500 /<br>1:500<br>1:250	Western blot /<br>Immunoprecipitation<br>Proximity ligation assay
Anti-Flag	Sigma	M2	1:20000	Western blot
Anti-Flag M2 affinity gel	Sigma	M2		Immunoprecipitation
Anti-Actin	Millipore	C4	1:10000	Western blot
Anti-p63	Abcam	Ab124762	1:2000	Western blot

Supplementary Table S2 Primer list

Species	Primer	Sequence
Mouse	*Gapdh*-F	5’-TGTCAAGCTCATTTCCTGGT-3’
	Gapdh-R	5’-TAGGGCCTCTCTTGCTCAGT-3’
	Mcp1-F	5’-AACTCTCACTGAAGCCAGCTCT-3’
	Mcp1-R	5’-CGTTAACTGCATCTGGCTGA-3’
	Il1β-F	5’-GGGCCTCAAAGGAAAGAATC-3’
	Il1β-R	5’-TTGCTTGGGATCCACACTCT-3’
	Il6-F	5’-GACAAAGCCAGAGTCCTTCAG-3’
	Il6-R	5’- GTCTTGGTCCTTAGCCACTC-3’
	Tnf-F	5’-CCACCACGCTCTTCTGTCTA-3’
	Tnf-R	5’-GAGGCCATTTGGGAACTTCT-3’
	Tgfβ-F	5’-CAACAATTCCTGGCGTTACC-3’
	Tgfβ-R	5’-AGCCCTGTATTCCGTCTCCT-3’
	Acta2-F	5’-TGCTGACAGAGGCACCACTG-3’
	Acta2-R	5’-CCAGAGGCATAGAGGGACAG-3’
	Col1α1-F	5’-TGGTGAGACTGGTCCTGCT-3’
	Col1α1-R	5’-CTGTCACCTTGTTCGCCTG-3’
	Col3α1-F	5’-GTAAGCACTGGTGGACAGAT-3’
	Col3α1-R	5’-AGCTGCACATCAACGACATC-3’
	Vegfa-F	5’-GCAGATGTGACAAGCCAAGC-3’
	Vegfa-R	5’-CGTCTTTCCGGTGAGAGGTC-3’
	Cox2-F	5’-TCCATTGACCAGAGCAGAGA-3’
	Cox2-R	5’-CATCGATGTGACTGTAGAGG-3’

Supplementary Table S2 Primer list (-continued)

Species	Primer	Sequence
	NFκb1-F	5'-TAAGCAGAAGCATTAACTTCTCTGGA-3'
	NFκb1-R	5'-CCTGCTTCTGTCTCTAGGAGAGTA-3'
Mouse/Human	*p53*-F	5'-AACGCTTCGAGATGTTCCG-3'
	p53-R	5'-CTTCTTGGTCTTCAGGTAG-3'
Human	*VEGFA*-F	5'-ATTGACAGCAGCGGGCA-3'
	VEGFA-R	5'-AGTTCGAGGAAAGGGAAAGGG-3'
	COX2-F	5'-CCCACAGTCAAAGATACTCAGG-3'
	*COX*2-R	5'-CCCACAGCAAACCGTAGATG-3'
	NFκB1-F	5'-AGTAAACCGGAACTCTGGGAG-3'
	NFκB1-R	5'-TTGTCGCACAGCAAGAAGAT-3'
	28S-F	5'-CGAATACAAGACCGTGAAAGC-3'
	28S-R	5'-GATAGGAAGAGCCGACATCG-3'
	S100A2-F*	5'-ACATCCTGGTCATCCTG-3'
	S100A2-R*	5'-GAATTCAGGGTCGGTCTGGGCAG-3'

* Both primers were used for genotyping transgenic mice.

Supplementary Figure S1 by Pan SC et al

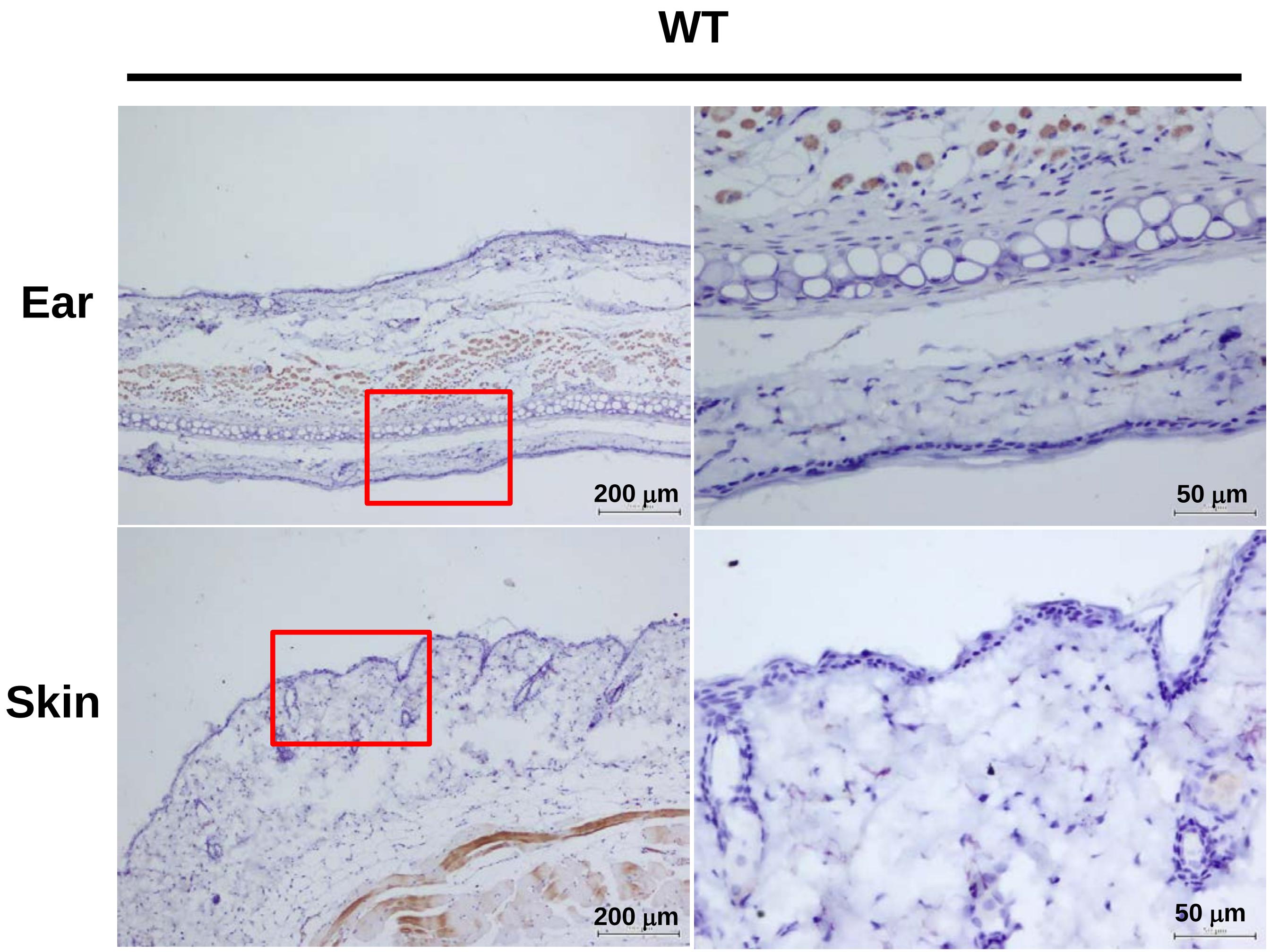

Supplementary Figure S2 by Pan SC et al

a

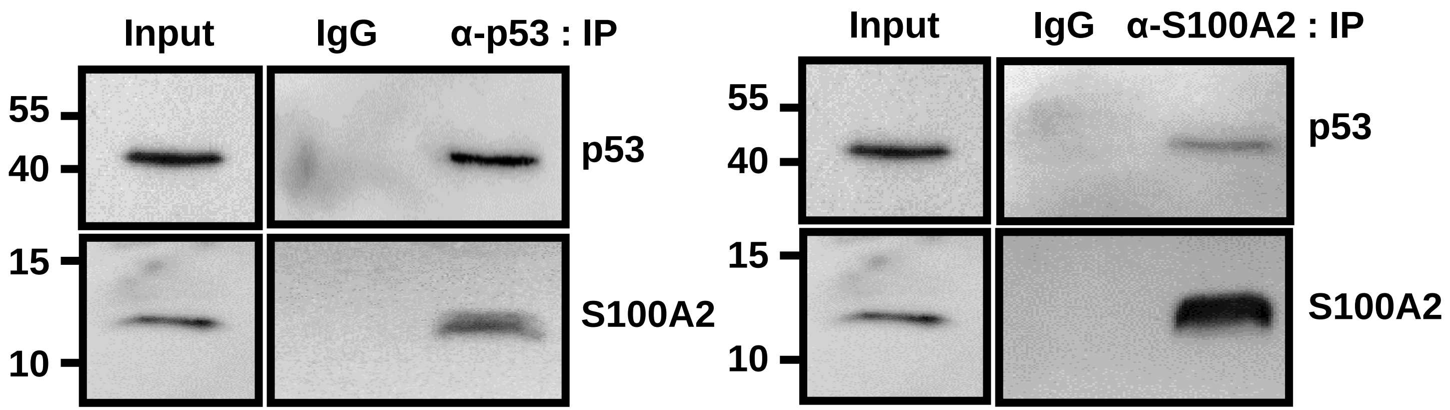

Supplementary Figure S2 by Pan SC et al

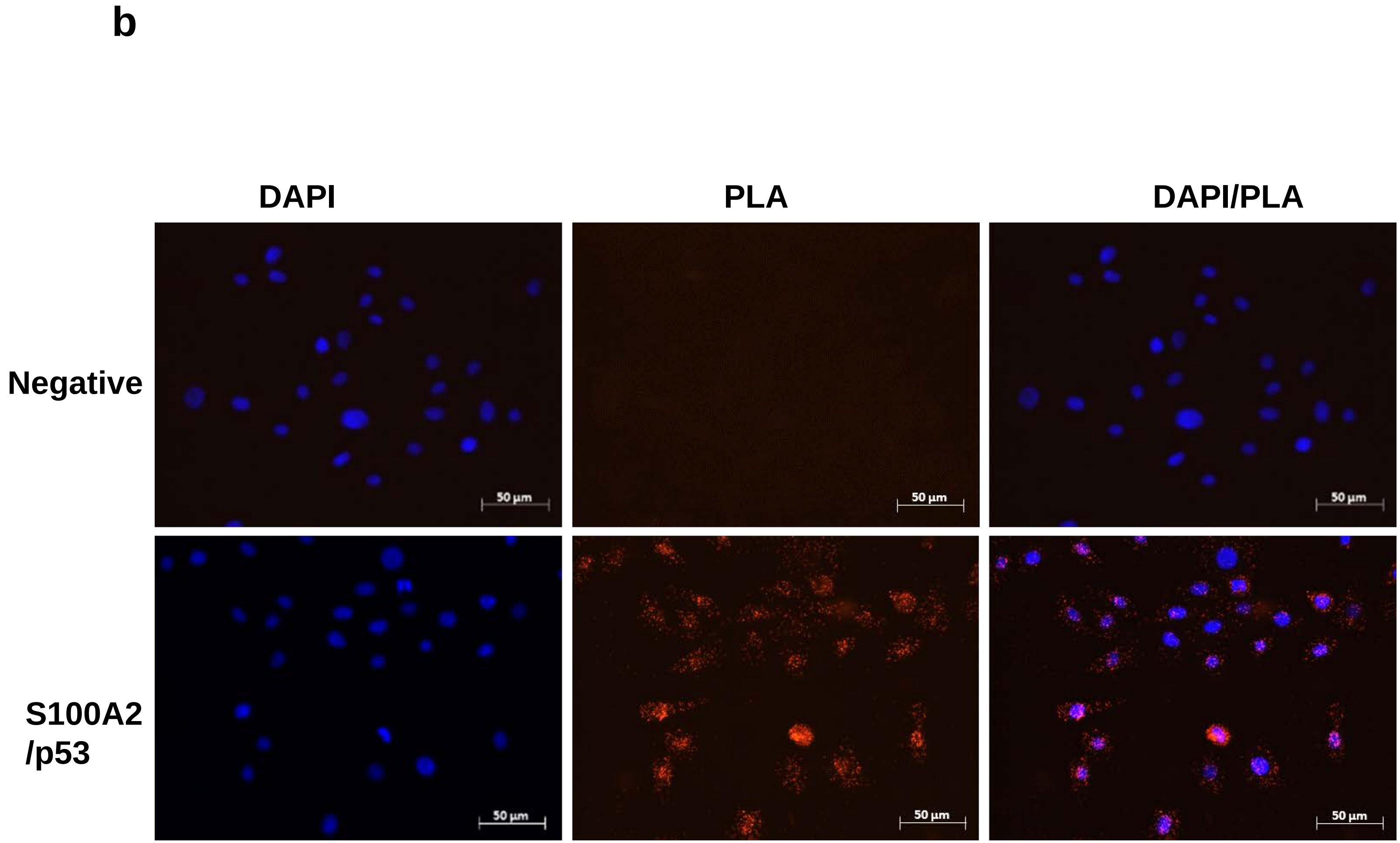

Supplementary Figure S3 by Pan SC et al

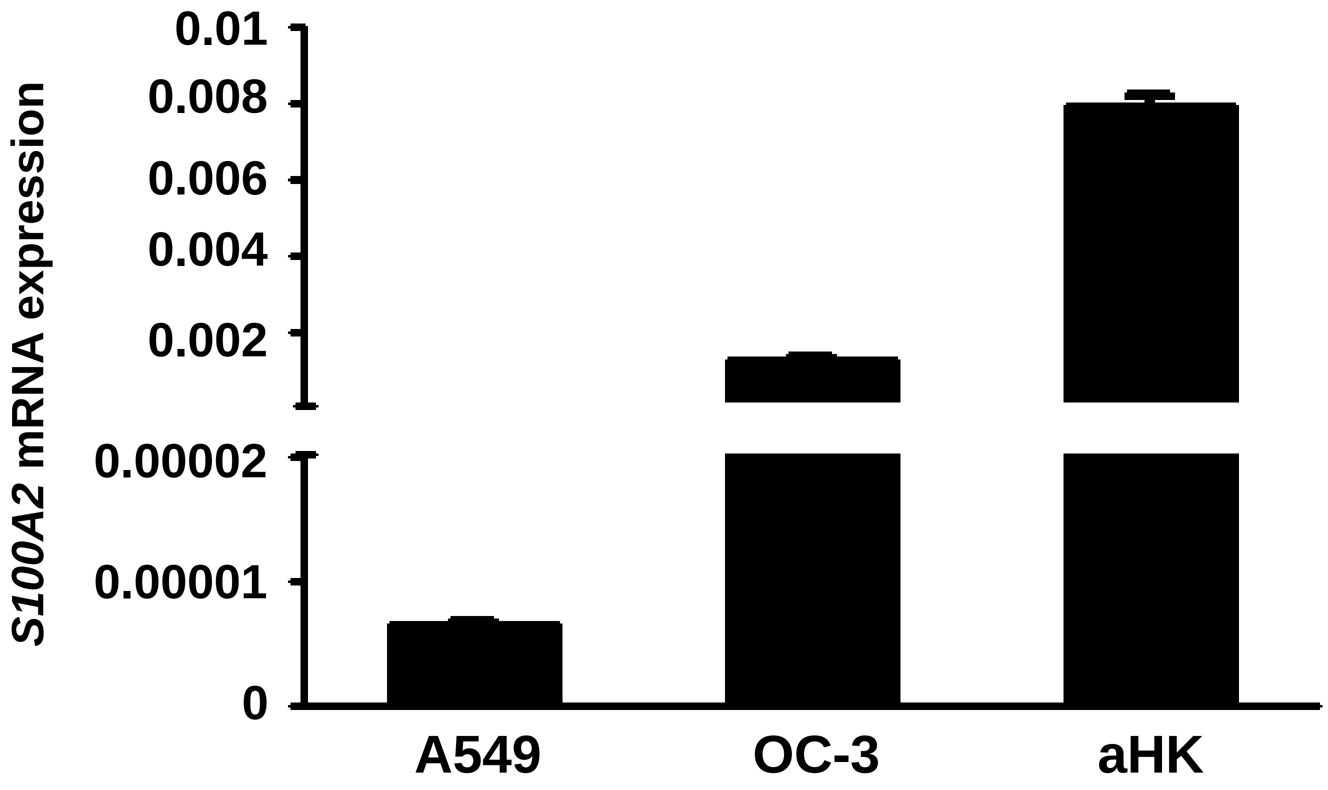

Supplementary Figure S4 by Pan SC et al

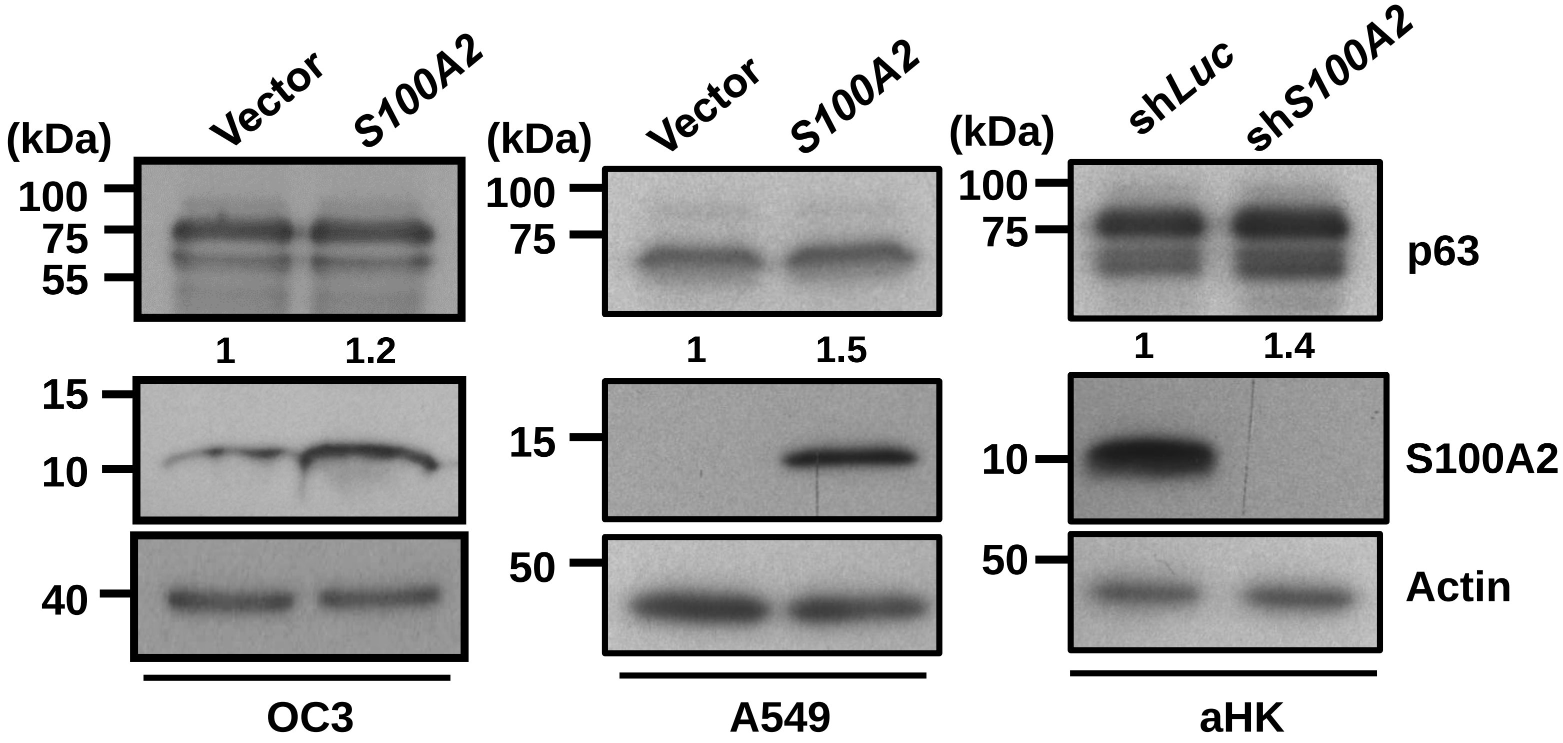

Supplementary Figure S5 by Pan SC et al

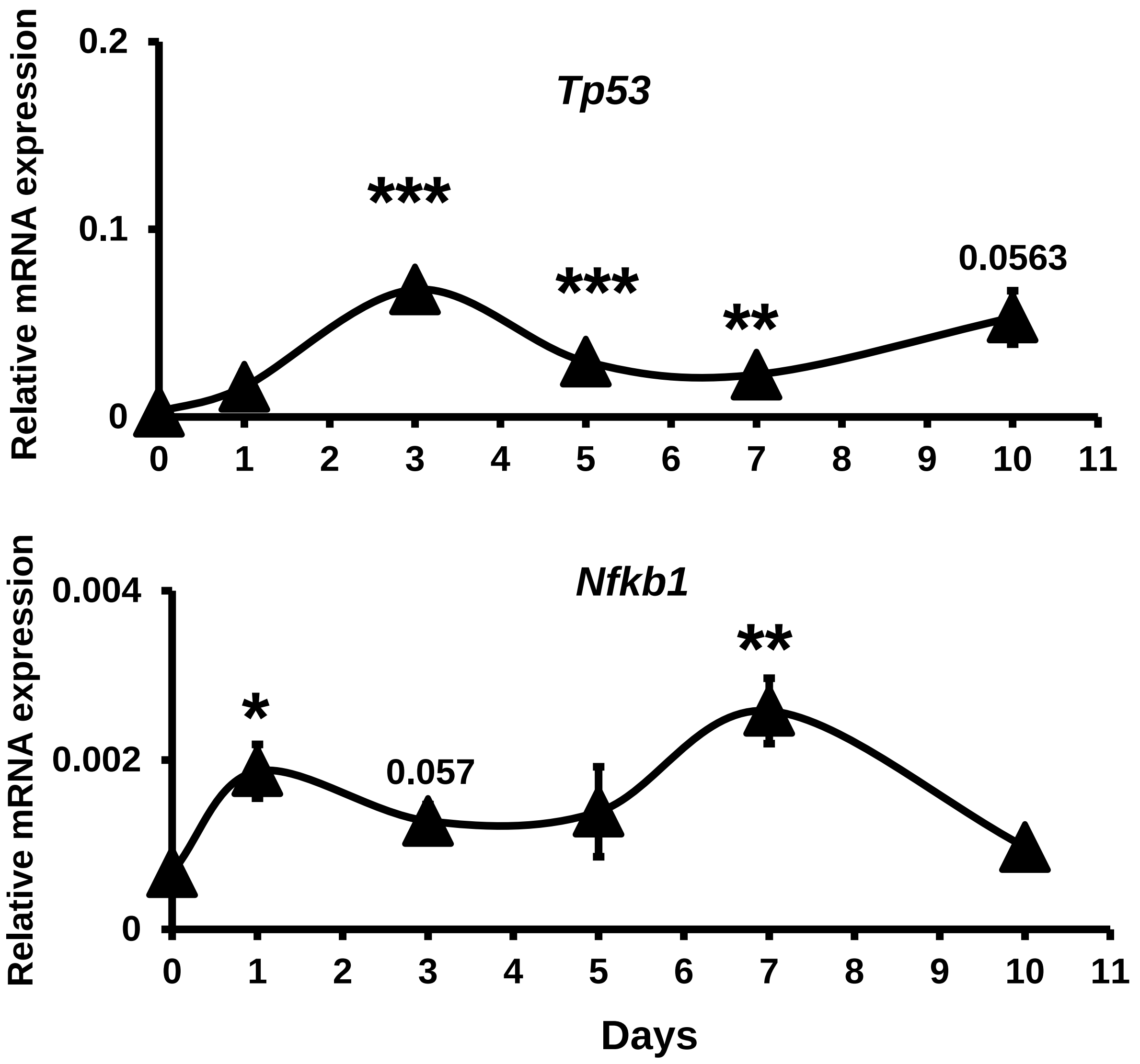

Uncropped Western blots for Figure 1d

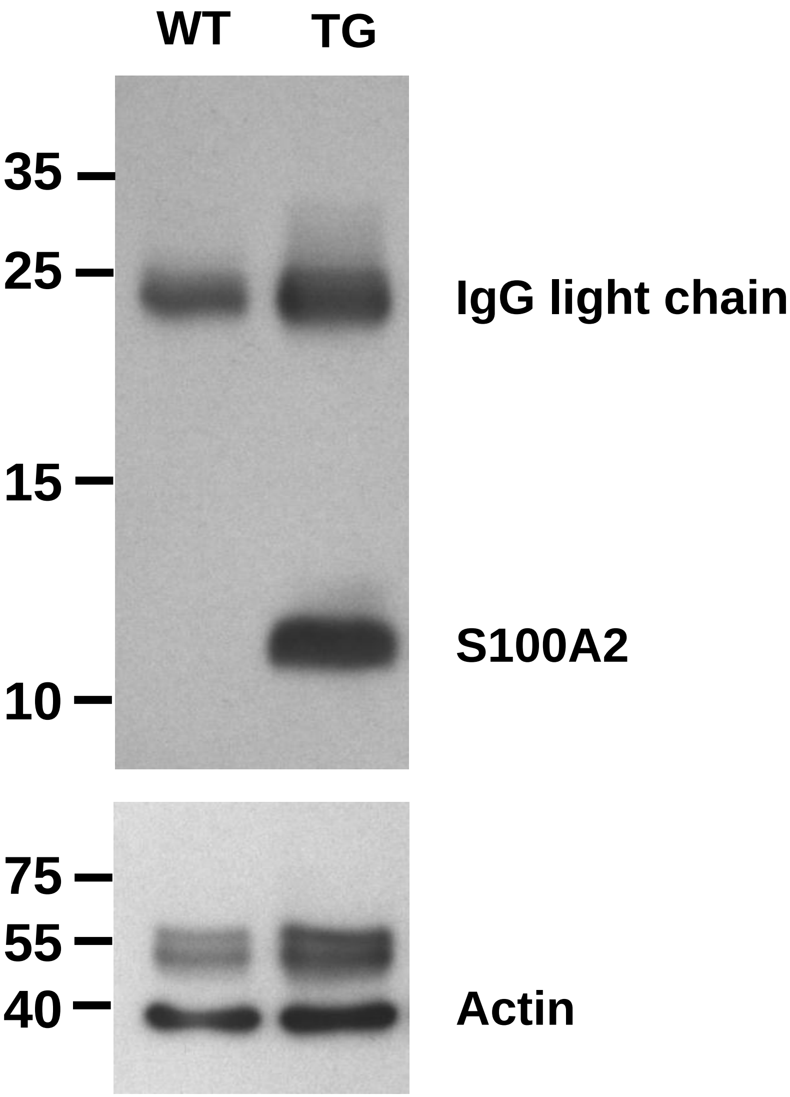

Uncropped Western blots for Figure 3a

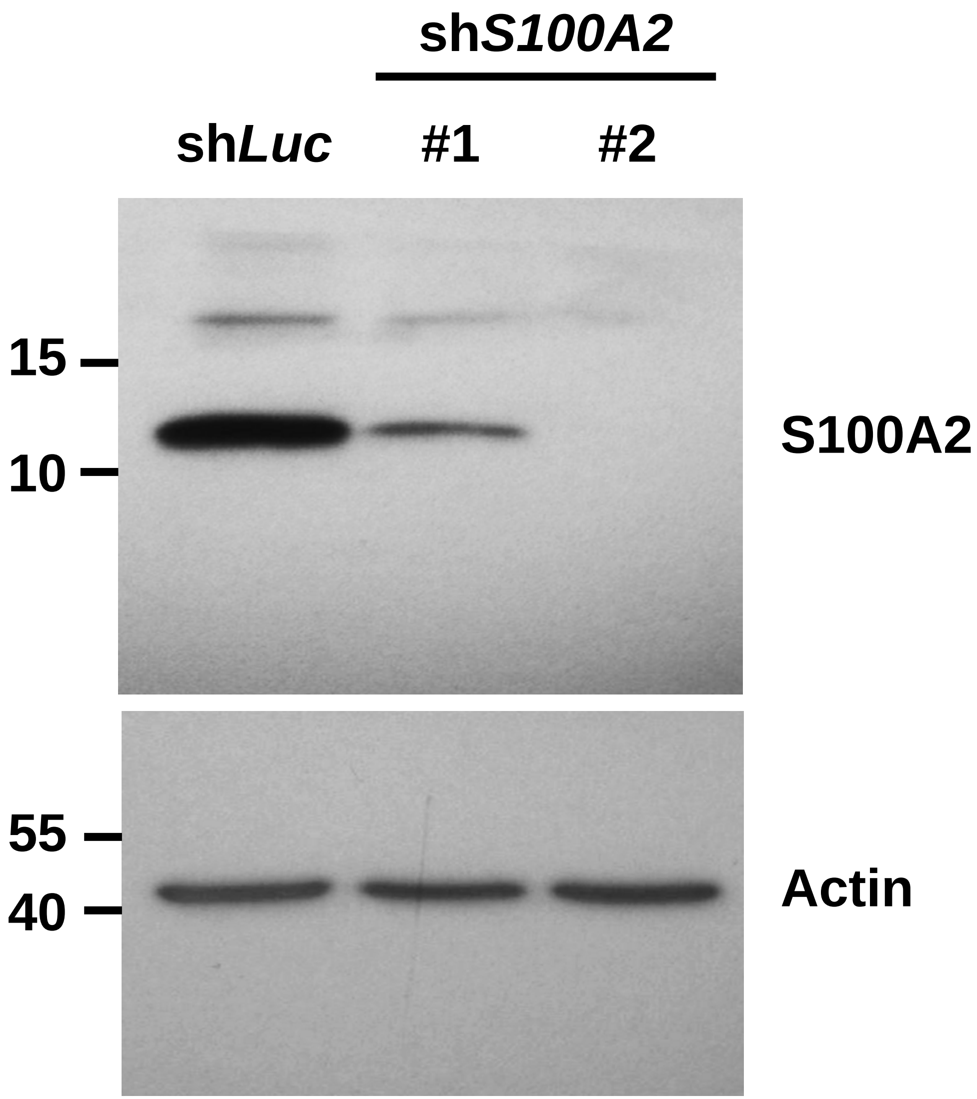

Uncropped Western blots for Figure 5a

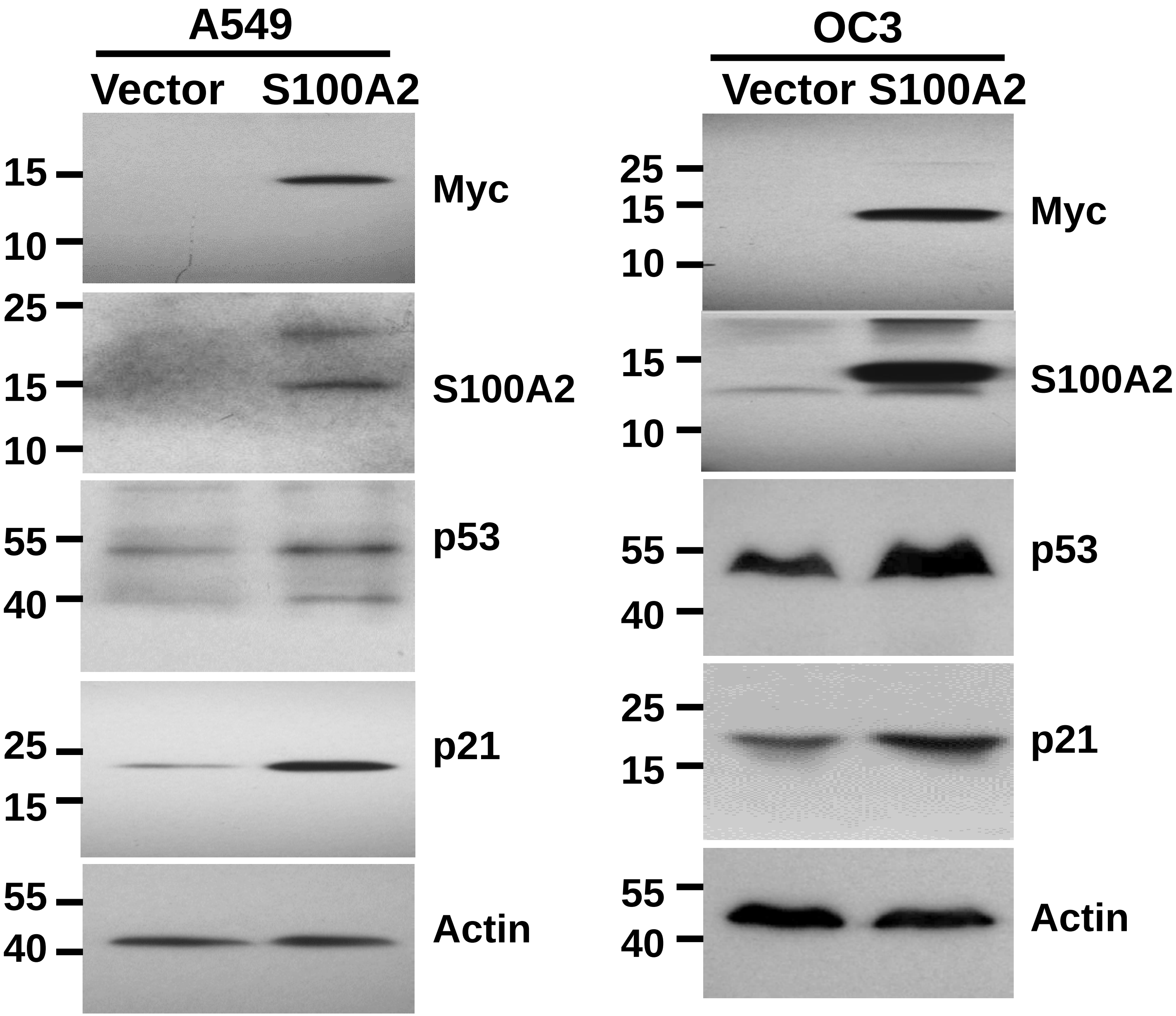

Uncropped Western blot for Figure 5c

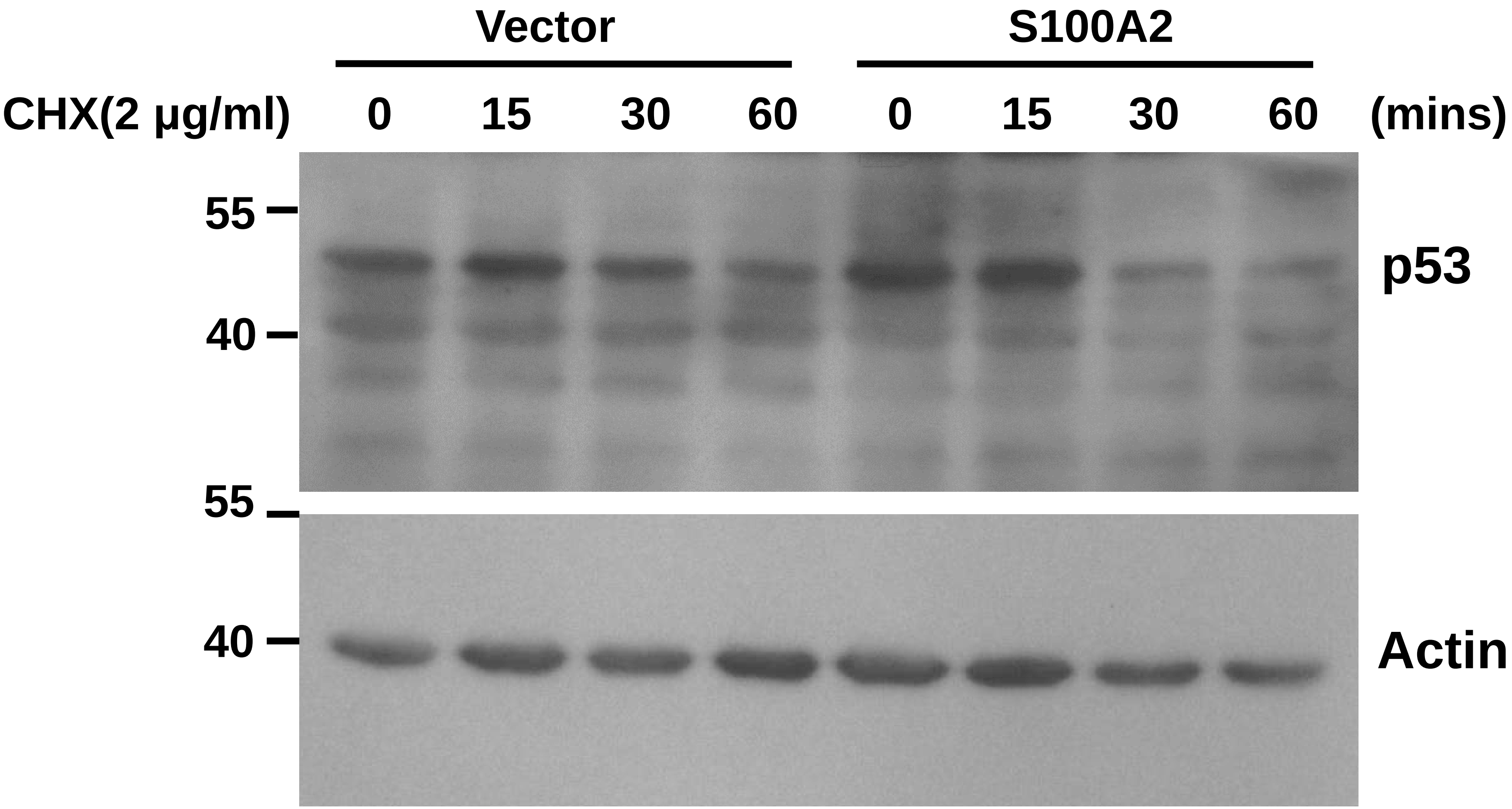

Uncropped Western blots for Supplementary Figure S2a

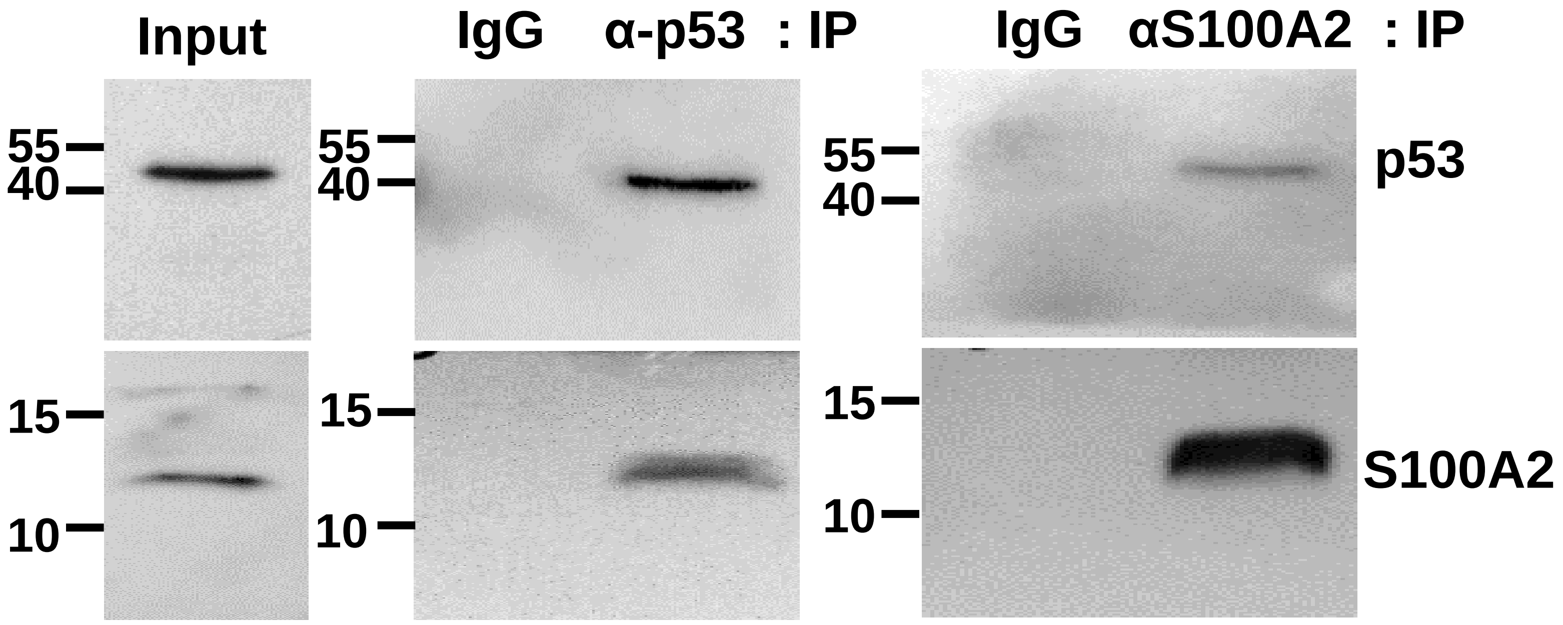

Uncropped Western blots for Supplementary Figure S4

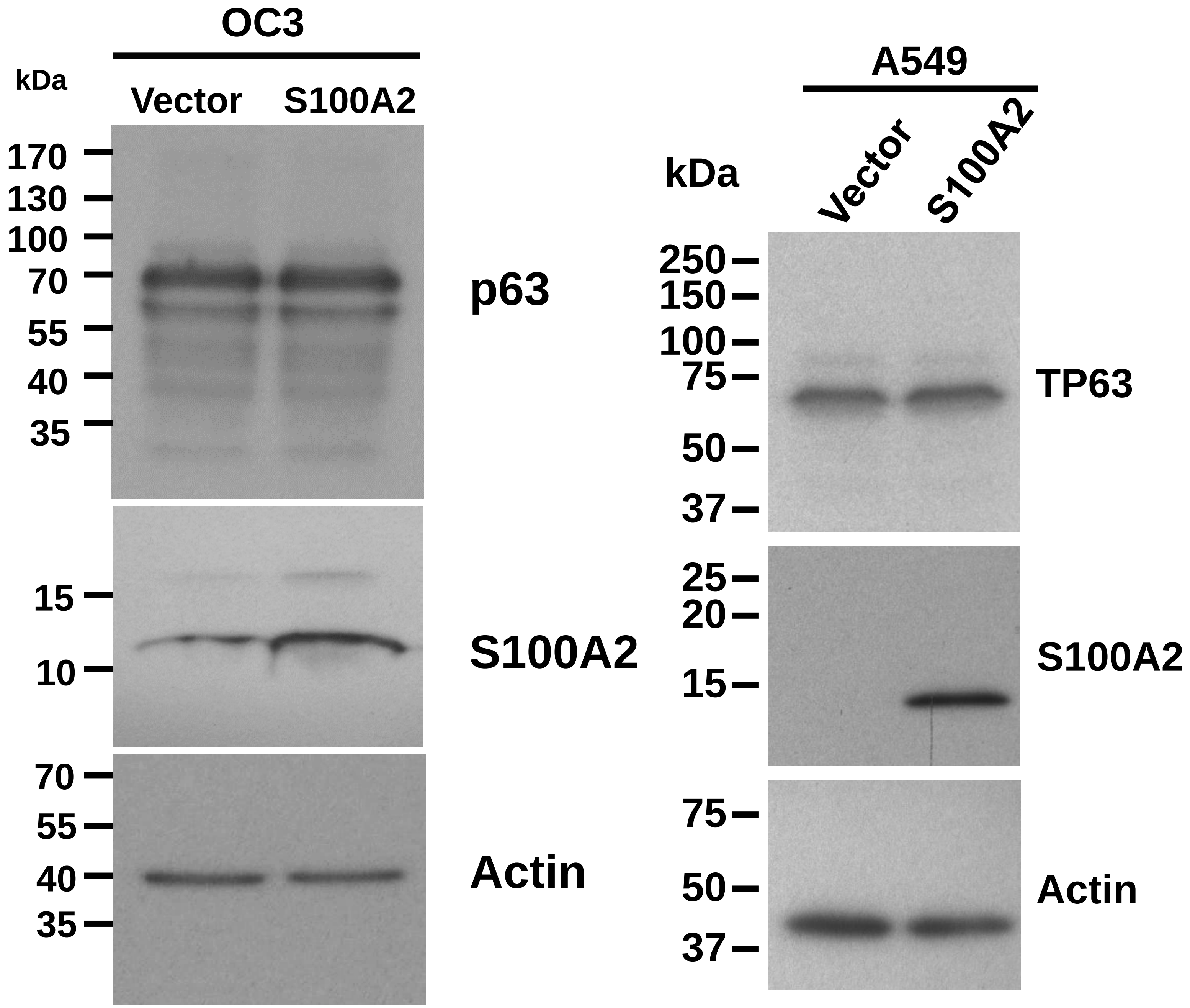

Uncropped Western blots for Supplementary Figure S4

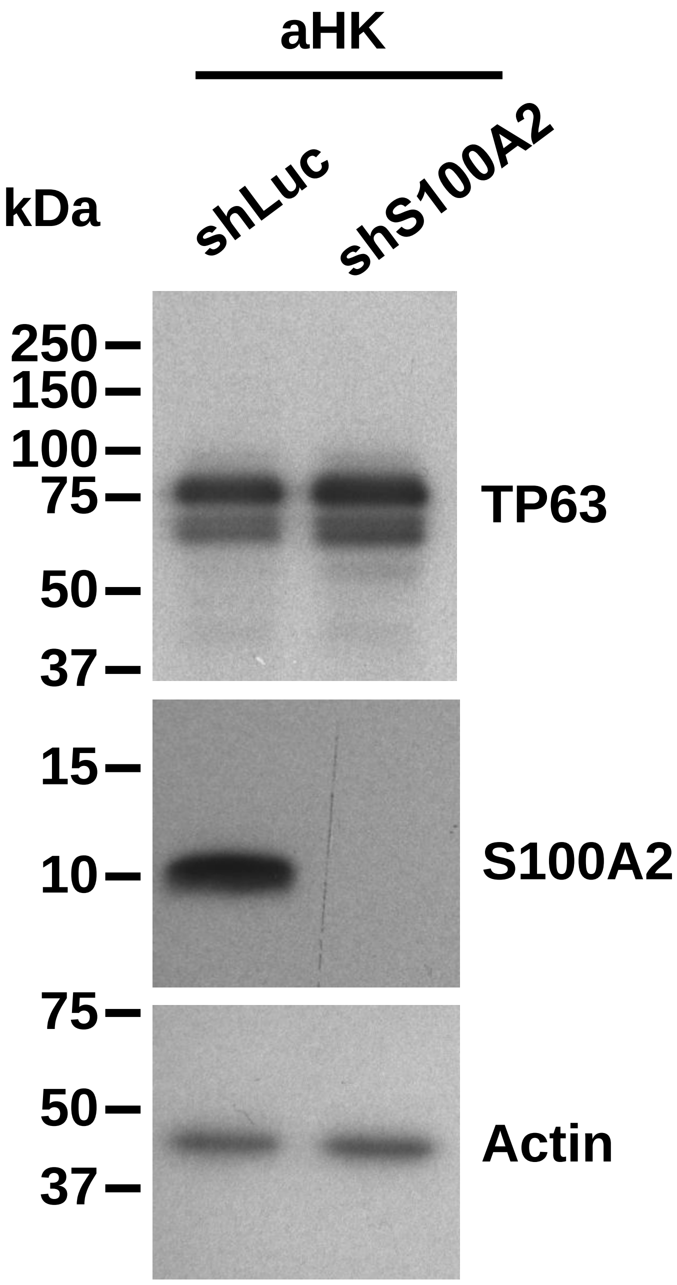

Supplement: Supplementary file 1 — Supplementary information [file 41598_2018_23697_MOESM1_ESM.pdf]
